# Drug Repurposing by Virtual Screening: Identification of New Already Approved ROCK Inhibitors as Promising Drugs to Target Neurodegeneration

**DOI:** 10.1021/acsomega.5c04340

**Published:** 2025-06-26

**Authors:** Lucas Silva Franco, Daniel Alencar Rodrigues, Gabriela Joras Baumart, Bárbara da Silva Mascarenhas de Jesus, Flávia Carvalho Alcantara Gomes, Lídia Moreira Lima, Carlos Alberto Manssour Fraga, Pedro de Sena Murteira Pinheiro

**Affiliations:** † Laboratório de Avaliação e Síntese de Substâncias Bioativas (LASSBio), Instituto de Ciências Biomédicas, 28125 Universidade Federal do Rio de Janeiro, 21941-902 Rio de Janeiro, Brazil; ‡ Instituto Nacional de Ciência e Tecnologia de Fármacos e Medicamentos (INCT-INOFAR), Instituto de Ciências Biomédicas, 28125 Universidade Federal do Rio de Janeiro, 21941-902 Rio de Janeiro, Brazil; § School of Pharmacy and Biomolecular Sciences (PBS), 8863 Royal College of Surgeons in Ireland, First Floor Ardilaun House Block B 111 St Stephen’s Green, Dublin 2 DO2 YN77, Ireland; ∥ Laboratório de Neurobiologia Celular, Programa de Pós-Graduação em Ciências Morfológicas, Instituto de Ciências Biomédicas, 28125 Universidade Federal do Rio de Janeiro, 21941-902 Rio de Janeiro, Brazil; ⊥ Programa de Pós-Graduação em Farmacologia e Química Medicinal, Instituto de Ciências Biomédicas, 28125 Universidade Federal do Rio de Janeiro, 21941-902 Rio de Janeiro, Brazil

## Abstract

ROCK kinases are key players in neurodegenerative diseases such as Alzheimer’s disease (AD), making them attractive therapeutic targets. In this study, we developed a pharmacophoric map of ROCK inhibitors and highlighted the key affinity sites in ROCK1/2 through molecular modeling. Virtual screening led to the identification of six approved drugs as ROCK inhibitors: ruxolitinib (**36**), baricitinib (**37**), ponatinib (**38**), tivozanib (**39**), nialamide (**40**), and tucatinib (**41**). Ruxolitinib (**36**) (*h*ROCK1 IC_50_ = 0.025 μM; *h*ROCK2 IC_50_ = 0.007 μM) and baricitinib (**37**) (*h*ROCK1 IC_50_ = 0.019 μM; *h*ROCK2 IC_50_ = 0.011 μM) showed the highest potency, while tivozanib (**39**) displayed 15-fold selectivity for ROCK2 over ROCK1. Molecular dynamics revealed that ruxolitinib (**36**) forms stable bidentate hydrogen bonds with the ROCK hinge region and has selectivity across the AGC kinase family. Biological assays confirmed ruxolitinib’s (**36**) safety in neuronal and glial cells and its ability to reduce C3 immunolabeling, a glial inflammation marker, in LPS-treated astrocytes. These findings not only highlight ruxolitinib (**36**) as a promising candidate for AD but also provide a structural basis for designing novel dual JAK-ROCK inhibitors and pave the way for further *in vitro* and *in vivo* studies. Moreover, the validated pharmacophoric map for ROCK inhibition highlights the identification of an affinity pocket that can be useful for the design of new ROCK inhibitors.

## Graphical abstract

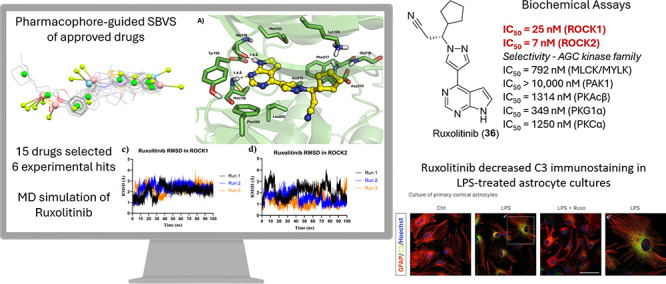

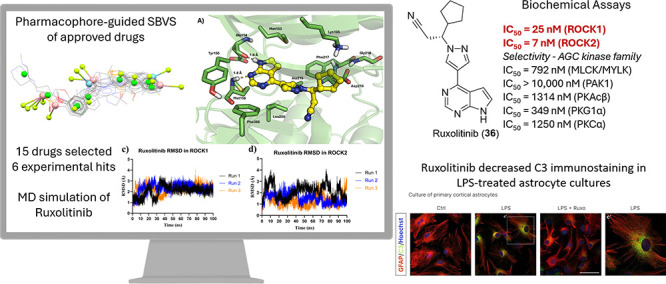

## 1 Introduction

Rho-associated protein kinases (ROCK) are ubiquitously expressed serine/threonine kinases and are the main effectors of the signaling cascade regulated by the small GTPase RhoA.^1^ These kinases participate in a wide variety of cellular processes including inflammation, contraction, cell adhesion, migration, and proliferation.^2^ There are two highly homologous isoforms of ROCK, ROCK1 and ROCK2, with 65% homology in their primary sequence, reaching 92% homology in the kinase domain.^3^ Both isoforms are widely expressed in several mammalian tissues.^3^

Recently, ROCK has been highlighted as an interesting target for the treatment of Alzheimer's Disease (AD).^4^ The RhoA/ROCK signaling pathway modulates the hydrolysis of the amyloid-β protein precursor (APP), which causes amyloid-β (Aβ) accumulation and increased neurotoxicity. *In vivo* studies have reported that APP is a substrate for ROCK1, and activation of ROCK1 enhances APP cleavage by β-secretase, increasing Aβ levels and promoting the pathogenesis of AD.^5^ Furthermore, inhibition of APP phosphorylation or inhibition of ROCK activity through knockdown or using an inhibitor improved learning and memory in APP/PS1 mice.^5^ Another study showed that the selective inhibition of ROCK2 suppressed the enzymatic activity of the BACE1 enzyme, decreasing Aβ production*in vivo*.^6^ It has also been shown that Aβ42 oligomers marginally increased ROCK1 and ROCK2 levels in neurons, but strongly induced phosphorylation of Lim kinase 1 (LIMK1), suggesting that Aβ42 activates ROCK.^7^ Depletion of ROCK1 or ROCK2 by RNAi suppresses endogenous Aβ40 production in neurons, and Aβ40 levels have been reduced *in vivo*.^7^ Numerous studies have also shown that ROCK inhibition reduces pTau levels. The drug Fasudil (**2**) decreased the levels of Tau phosphorylation in primary cultures of mouse hippocampal neurons.^8^ Studies conducted both *in vitro* and *in vivo* have demonstrated that ROCK inhibitors can decrease the levels of phosphorylated Tau (pTau) and oligomeric Tau. Additionally, these inhibitors facilitate the degradation of Tau by suppressing the activity of Tau kinases, such as GSK3β and Cdk5, and enhance autophagic and proteasome pathways crucial for Tau clearance.^9^ *In vivo* studies have shown that ROCK induces microglia activation, which leads to a neurotoxic phenotype and increases the expression of nitric oxide synthase and TNF-α, causing neurodegeneration.^10^ Other *in vivo* and *in vitro* studies have demonstrated that ROCK activation can regulate expression of the AT1 receptor through the NF-κB pathway, increasing neuroinflammation.^11^

Thus, ROCK inhibition is a new therapeutic strategy for the treatment of AD, since it affects the production of the main pathophysiological markers of AD, as well as being associated with the reduction of neuroinflammation. In addition, ROCK inhibitors show potential for the treatment of familial Cerebral Cavernous Malformation, Parkinson’s disease, Huntington’s disease, Multiple sclerosis, Amyotrophic lateral sclerosis, among other neurodegenerative diseases.^12–16^

To date, four ROCK inhibitors are approved for clinical use in different countries. Netarsudil (**1**) (^ROCK1^IC_50_ = 79 nM, ^ROCK2^IC_50_ = 16 nM)^17^ and Belumosudil (**4**) (^ROCK1^IC_50_ = 3000 nM, ^ROCK2^IC_50_ = 100 nM)^18^ are approved in the United States. Fasudil (**2**) (^ROCK1^IC_50_ = 940 nM, ^ROCK2^IC_50_ = 220 nM)^19^ is approved in Japan and China. Ripasudil (**3**) (^ROCK1^IC_50_ = 51 nM, ^ROCK2^IC_50_ = 19 nM)^20^ is approved in Japan (Figure 1). Netarsudil (**1**) and Ripasudil (**3**) were approved for the treatment of glaucoma and ocular hypertension, while Fasudil (**2**) was approved for the treatment of cerebral vasospasm, and Belumosudil (**4**) was approved for the treatment of graft-versus-host disease.^20–23^ These inhibitors interact at the ATP binding site of the ROCK kinase domain.^20–23^ The heterocyclic ring in their structures performs hydrogen bond interactions with the hinge region in the adenine binding pocket, essential for competing with ATP binding, as can be seen by Fasudil’s (**2**) interaction mode in ROCK1 (PDB: 2ESM) (Figure 1).^24^

**Figure 1:**
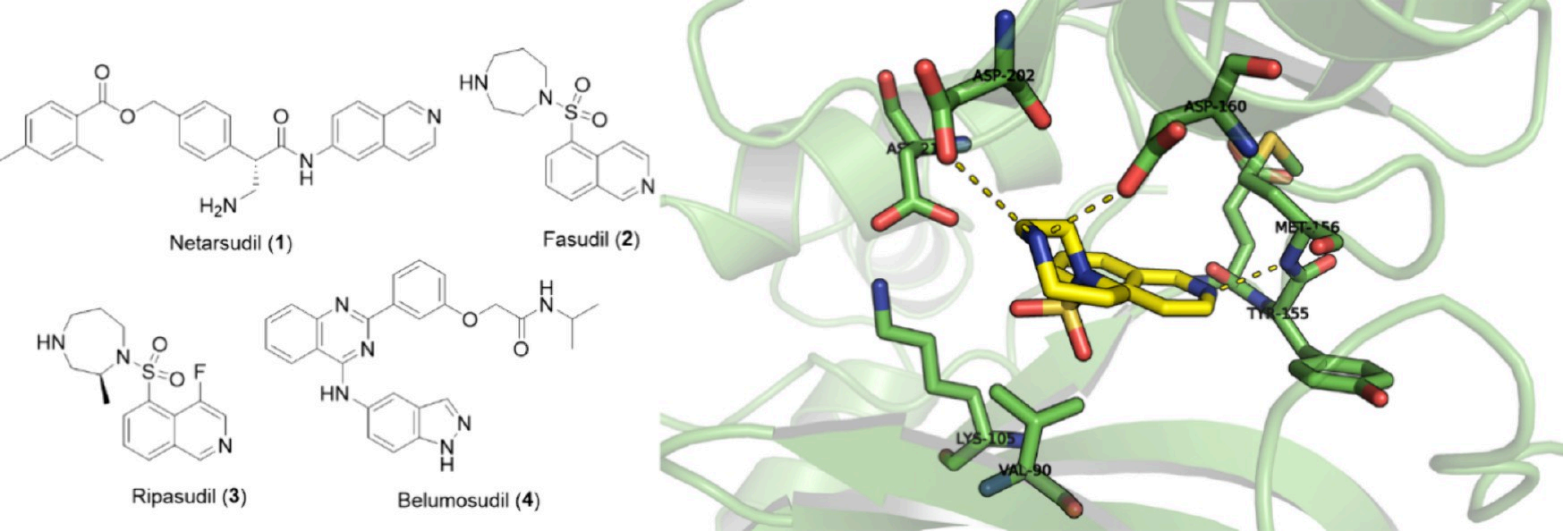
Approved ROCK inhibitors and the interaction mode of fasudil (**2**) on ROCK1 (PDB: 2ESM).^24^

Despite several classes of inhibitors being reported in the literature, there is a need to discover new inhibitors with suitable potency and physicochemical properties for the development of drugs for the treatment of neurodegenerative diseases. In this context, drug repurposing, also known as drug repositioning or drug refiling, is an interesting strategy related to the process of identifying new therapeutic uses for existing drugs. This approach has gained significant attention in recent years due to its potential to expedite the drug development process and reduce costs compared with traditional drug discovery methods. By repurposing existing drugs, exploration of their known safety profiles and pharmacokinetic properties can accelerate the clinical translation of these drugs to new therapeutic indications.^25–27^

Our objective of this work is to accelerate the identification of useful drugs for the treatment of AD based on drug repurposing through the identification of new ROCK inhibitors already in the market. In addition, we hope to characterize the pharmacophoric features necessary for designing potent ROCK1/2 inhibitors using ligand-based and structure-based complementary strategies to help drug development within ROCK inhibitor discovery projects.

Initially, we performed a chemical space analysis of known ROCK inhibitors to better understand their current structural and physicochemical features. Next, we analyzed crystallographic structures deposited in the Protein Data Bank to evaluate how these chemical features interact within the ROCK binding sites and to construct a preliminary pharmacophoric map. Based on these findings, we developed and optimized a virtual screening protocol guided by pharmacophore modeling for ROCK inhibition. Subsequently, we selected candidate compounds and validated their biological activity in vitro by using different neural cell lines and primary cultures. Ultimately, this integrative approach led to the identification of six approved drugs—originally developed for other diseases and molecular targets—that exhibit potent ROCK inhibitory activity and thus represent promising candidates for drug repurposing in neurodegenerative disorders.

## 2 Results and Discussion

### 2.1 Chemical Space Analysis of ROCK Inhibitors

We evaluated the chemical space of potent ROCK1 and ROCK2 ligands (*K*_i_ or *K*_d_ ≤ 100 nM) employing chemometric tools, such as principal component analysis (PCA) and multidimensional scaling (MDS).^28–30^ Compound selection was performed in the ChEMBL database. Chemical spaces of ROCK1/2 ligands were analyzed using the KNIME platform.^31^ Smiles strings of the ligands obtained from ChEMBL were transformed to ECFP_6 chemical fingerprints, which encode molecular features up to a bond diameter of 6, offering a suitable description of local atom environments and larger substructural patterns for ROCK inhibitors.^32,33^ Z-score normalization was applied to bit vector integers to standardize feature scales, ensuring balanced contributions to PCA. Principal component analysis reduced the chemical information to 2 dimensions, which were then rescaled to a 0–1 range (minimum-maximum normalization) to equalize their influence in clustering. The optimal number of clusters was determined by calculating the mean silhouette coefficient (testing *k* = 3–30), a metric that evaluates cluster cohesion and separation. This approach objectively identified the best-performing clustering algorithm. Three clustering algorithms were tested: hierarchical clustering, *k*-means, and *k*-medoids (Table S1). Hierarchical clustering was selected for ROCK1 ligands due to its superior mean silhouette score (0.69), while *k*-means was chosen for ROCK2 ligands. Representative compounds for each cluster were identified using *k*-medoids, which selects the most central actual molecule (medoid) per cluster. For both data sets (Figure 2), the presence of densely populated clusters of molecules was observed for ROCK1 (purple cluster, 159 molecules) (Figure 2A) and for ROCK2 (red cluster, 199 molecules) (Figure 2B). Sparse data points that represented peptide derivatives and derivatives of the pan-kinase inhibitor staurosporine were also detected. In order to enable the selection of all staurosporine derivatives in ROCK1 hierarchical clustering, the number of clusters was raised to five, which still had the highest mean silhouette score compared with other methods (0.69).

**Figure 2:**
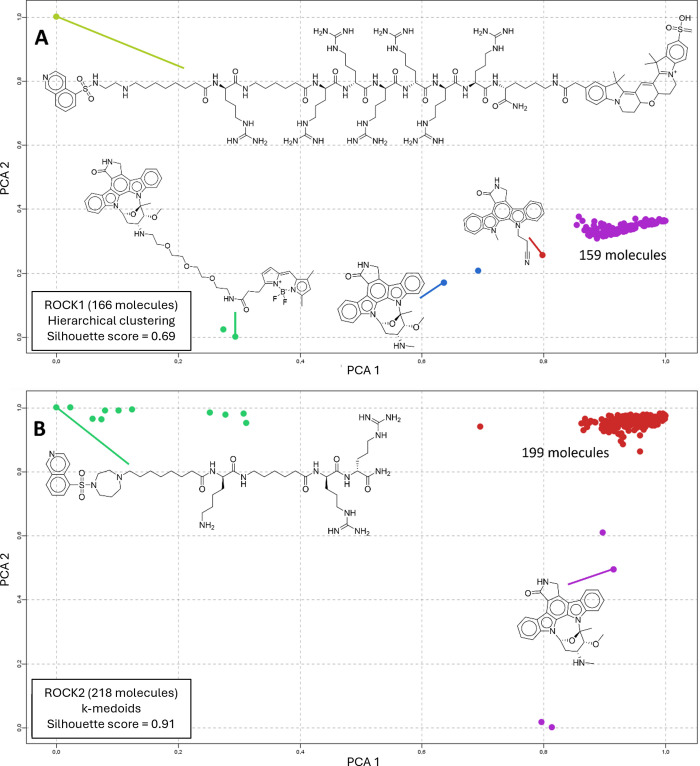
PCA applied to molecular descriptor ECFP_6 of selected ROCK1 (a) and ROCK2 (b) ligands. Compounds were clustered using hierarchical clustering (ROCK1) and *k*-means (ROCK2) methods, and the main representative compounds were determined using the *k*-medoids method within each cluster.

The most populated clusters of each data set are composed mainly of nitrogen heterocyclic compounds (Figure 2), which were further analyzed using MDS for dimensionality reduction. MDS projects the data, aiming to preserve the pairwise distances between the data points, while PCA projects the data by finding the directions of maximum variance.^29,30^ It is suggested that MDS produces a space where proximity better reflects structural similarity.^34^ Therefore, a distance matrix was obtained from pairwise Tanimoto similarity based on molecular fingerprints of each data set. The distance matrix was used as input for dimensionality reduction, which generated two dimensions employing MDS.

Similarly, to the first clustering step, mean silhouette coefficient calculation was performed for a range of 3–30 clusters. The maximum mean silhouette score was used to select the optimal number of clusters. Hierarchical clustering, *k*-means, and *k*-medoids methods were employed (see Supporting Information, Table S2). The maximum mean silhouette score indicated that *k*-means had better performance for both ROCK1/2 data sets. For the ROCK1 data set, the optimal number of clusters was 4, and for the ROCK2, the optimal number was 6. The *K*-medoids algorithm was employed to assign the main representative chemotypes of each cluster (Figure 3).^35^

**Figure 3:**
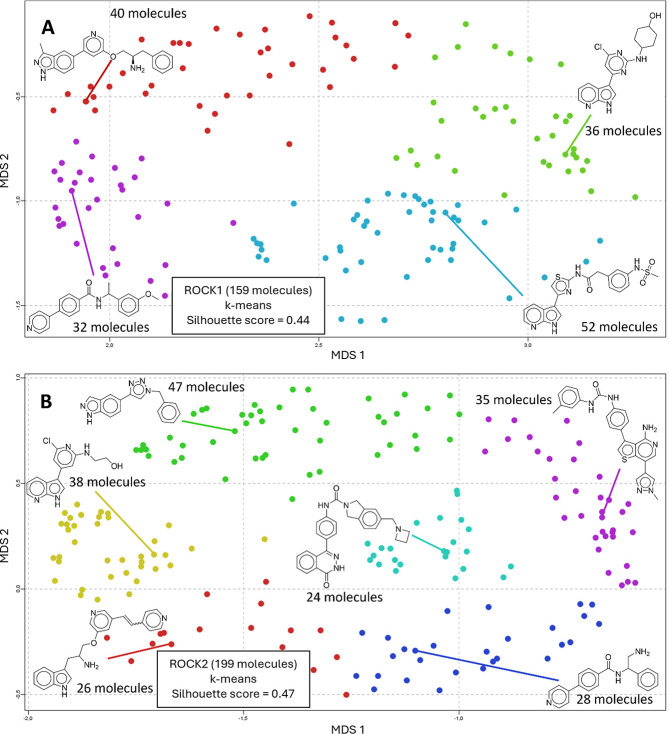
MDS applied to molecular descriptor ECFP_6 of the main cluster of ROCK1 (a) and ROCK2 (b) ligands. Compounds were clustered using the *k*-means method, and main representative compounds were determined using the *k*-medoids method within each cluster.

Clustering analysis indicated that all representative molecules of both data sets contain nitrogen heterocyclic derivatives, which is a common motif for kinase inhibitors.^36^ For ROCK1, the 7-azaindole subunit is present in representative compounds of two clusters (green and blue, Figure 3A). Additionally, the pyridine ring is present in representative molecules of the remaining two clusters (red and purple, Figure 3A), and the indazole ring is present in the representative molecule of the red cluster (Figure 3A). Common nitrogen heterocyclic motifs are present in the ROCK2 data set. 7-azaindole (yellow cluster, Figure 3B), 4-phenyl-pyridine (blue cluster, Figure 3B), and indazole (green cluster, Figure 3B) rings are also present in ROCK2 representative ligands. However, some additional structural patterns are also observed, such as 4-amino-thieno­[3,2-*c*]­pyridine (purple cluster, Figure 3B), indole (red cluster, Figure 3B), and phthalazinone (cyan cluster, Figure 3B).

### 2.2 Analysis of ROCK1/2 Crystal Structures

Beyond the chemical space analysis of ROCKi, the analysis of the ATP binding site of ROCK1 and ROCK2 allows a better understanding of their shape and identification of adjacent subcavities. Ultimately, the data can be explored in the design and optimization of known inhibitors to gain potency and selectivity across the kinome.

ROCK1 and ROCK2 are even more similar when comparing their ATP binding sites (Figure 4). Evaluation of crystallographic structures of ROCK1 (PDB: 6EW9)^37^ and ROCK2 (PDB: 6ED6)^37^ unambiguously states that they have 100% identity in the ATP binding site. All selected residues in the binding site resulted in perfect alignment (Figure 4). The overall shape is quite the same as can be visualized through the overlay of ROCK1 and ROCK2 binding sites, with an RMSD value of 0.885 Å (Figure 4). In addition, the values of the solvent accessible surface area of the respective binding sites are similar, 3830 Å^2^ in ROCK1 and 3881 Å^2^ in ROCK2.^38–41^

**Figure 4:**
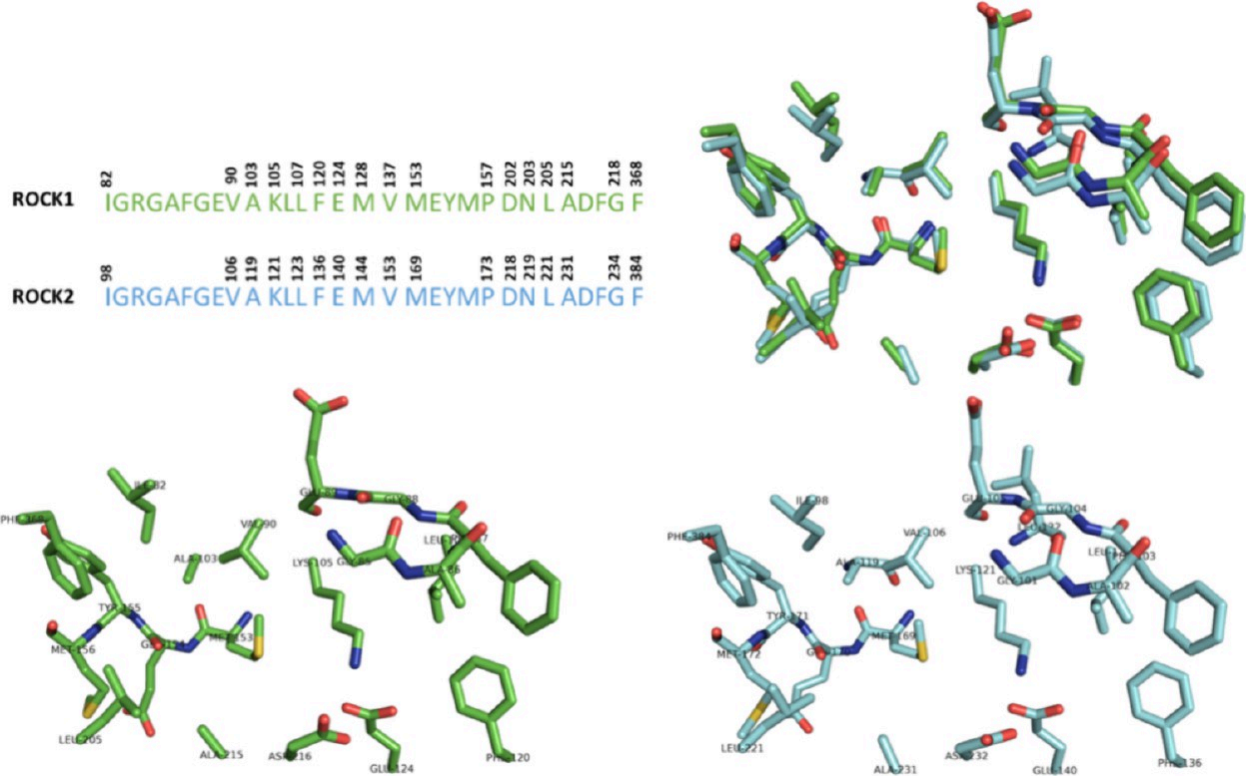
ATP binding site alignment of ROCK1 and ROCK2. ROCK1 (PDB: 6EW9), carbons in green. ROCK2 (PDB: 6ED6), carbons in cyan.

The binding site of ROCK is organized in the form of a straight channel, which may be divided in 4 main regions: the *hinge segment* is the deepest area in the cavity; the *glycine-rich loop*, which acts as a flexible lid; the *gatekeeper region*, which limits the access to hydrophobic back pockets in the catalytic spine; and the *catalytic region*, comprising the catalytic lysine residue, activation loop, and other phosphate-anchoring residues.

Specific residues appear to play a critical role in small-molecule recognition. Notably, Glu154 and Met156 in ROCK1 and Glu170 and Met172 in ROCK2 are located in the hinge region and are commonly involved in interactions with ligands. Additional key residues include those from the Gly-rich loop—Gly83 to Gly88 in ROCK1 and Gly99 to Gly104 in ROCK2—and the catalytic lysine (Lys105 in ROCK1 and Lys121 in ROCK2) (Figure 5A,B), which are essential for maintaining the structural conformation of the binding site and facilitating ligand interaction.

**Figure 5:**
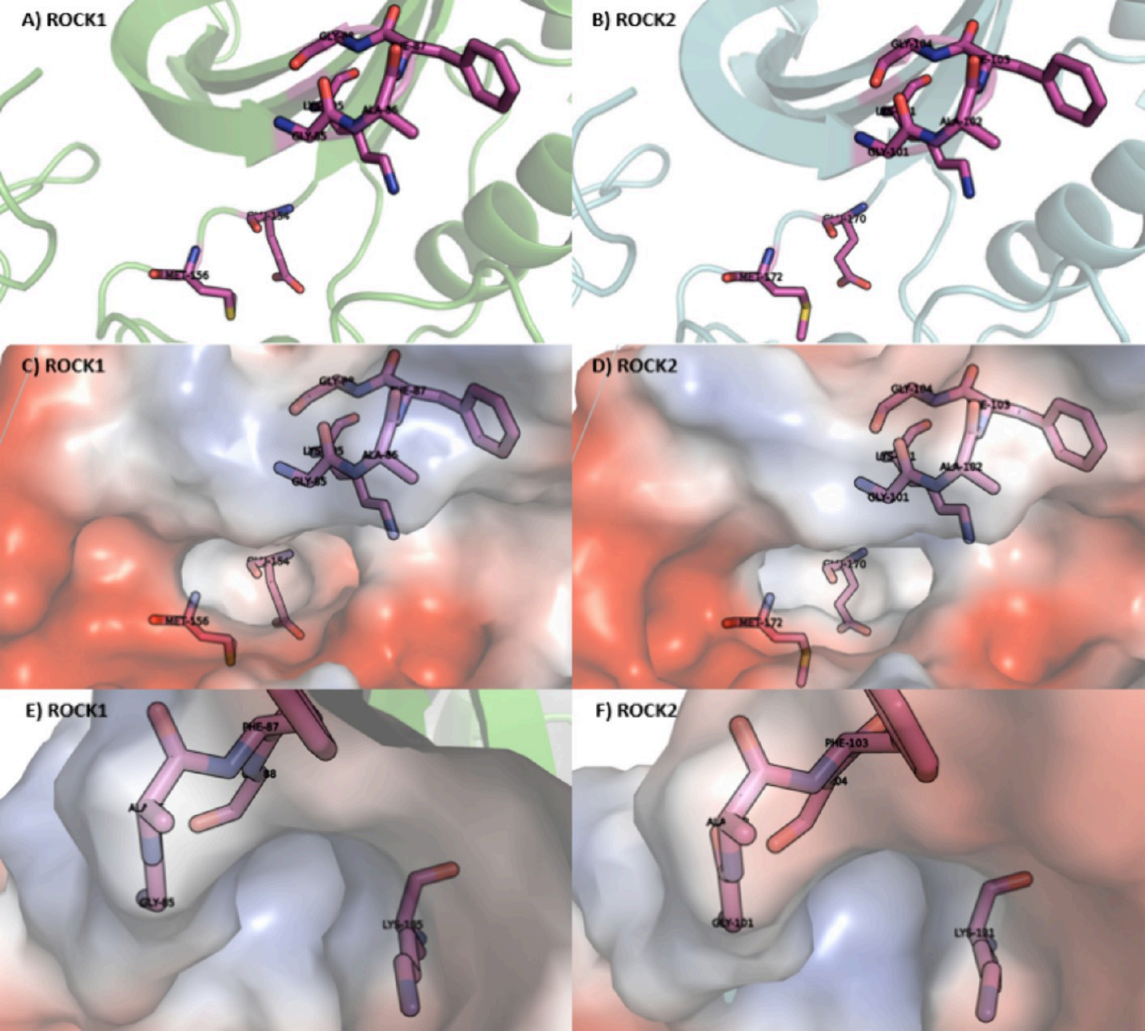
ATP binding site analysis of ROCK1 (A, C, and E) and ROCK2 (B, D, and F).

Furthermore, we identified a pocket formed between the Gly-rich loop and the catalytic lysine that is present in all crystallographic structures of ROCK1 and ROCK2 (Figure 5E,F). Despite its consistency, this pocket has not been emphasized in the literature as a relevant feature for structure-based drug design. Located at the solvent-exposed end of the binding site channel, orthogonal to the hinge motif, this pocket may significantly influence ligand affinity (Figure 5C–F). For this reason, we refer to it throughout this work as the “ROCK affinity pocket”.

### 2.3 Pharmacophoric Mapping of ROCK Inhibitors

On the basis of chemical space analysis, we started a protocol for the identification of the pharmacophoric map for the design of potent ROCKi, considering the entire chemical space of inhibitors. First, we selected the crystallographic structures of human ROCK1/2-inhibitor complexes from PDB, which had no mutations or deletions on the ATP binding site. In addition, the complexed ligand was interacting with the ATP binding site and its determined activity, IC_50_ or *K*_i_, was in the low nanomolar range. The selected crystallographic structures, chemical structures, and their activities are reported in Table 1.

**Table 1:**
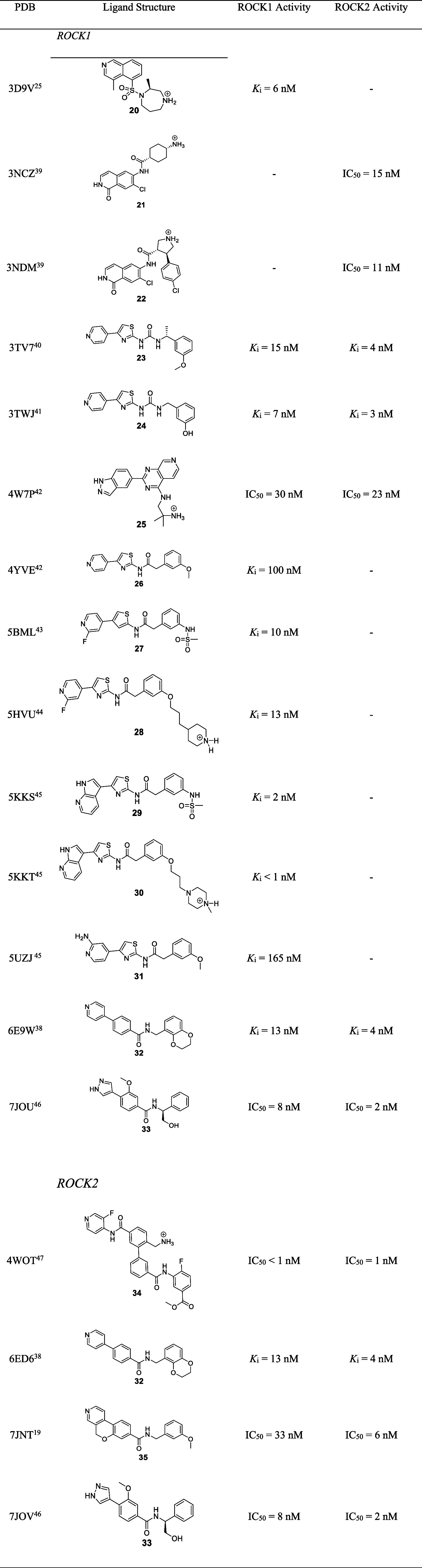
Selected PDB Structures for the Pharmacophoric Map Generation of ROCK1 and ROCK2 Inhibitors

In total, 14 crystal structures of ROCK1 and 4 crystal structures of ROCK2 were selected. The ligand scaffolds presented in Table 1 can be easily divided based on the hinge binder structural motif, in which there are phenylpyridine, phenylpyrazole, thiazolepyridine, isoquinoline, isoquinolone, indazole, and pyrrolopyridine-based motifs, in agreement with the chemical space analysis conducted in this work. These hinge binder motifs explore hydrogen bond interactions with the main chain of Met156 of ROCK1 and Met172 of ROCK2 through sp^2^ aromatic nitrogen in their structures. Compounds **5** and **6**, cocrystallized with ROCK1, explore additional hydrogen bond interactions of carbonyl oxygen in their isoquinolone scaffold. Moreover, it is possible to see that, in general, the ligands are based on bicyclic aromatic or biaromatic systems, followed by a benzylamide or benzylurea-based linker that breaks the systems’ planarity and a terminal aromatic ring, usually *meta*-substituted.^42–46^

The selected ligands were extracted from the complexes, protonated, and aligned in their bioactive conformation using Ligand Overlay from the CSD package.^47,48^ The used tautomeric and ionization states are indicated in Table 1. The analysis using Ligand Overlay revealed the main pharmacophoric points for interaction with the ATP binding site of both enzymes, resulting in two pharmacophoric maps, one for ROCK1 and one for ROCK2 (Figure 6).

**Figure 6:**
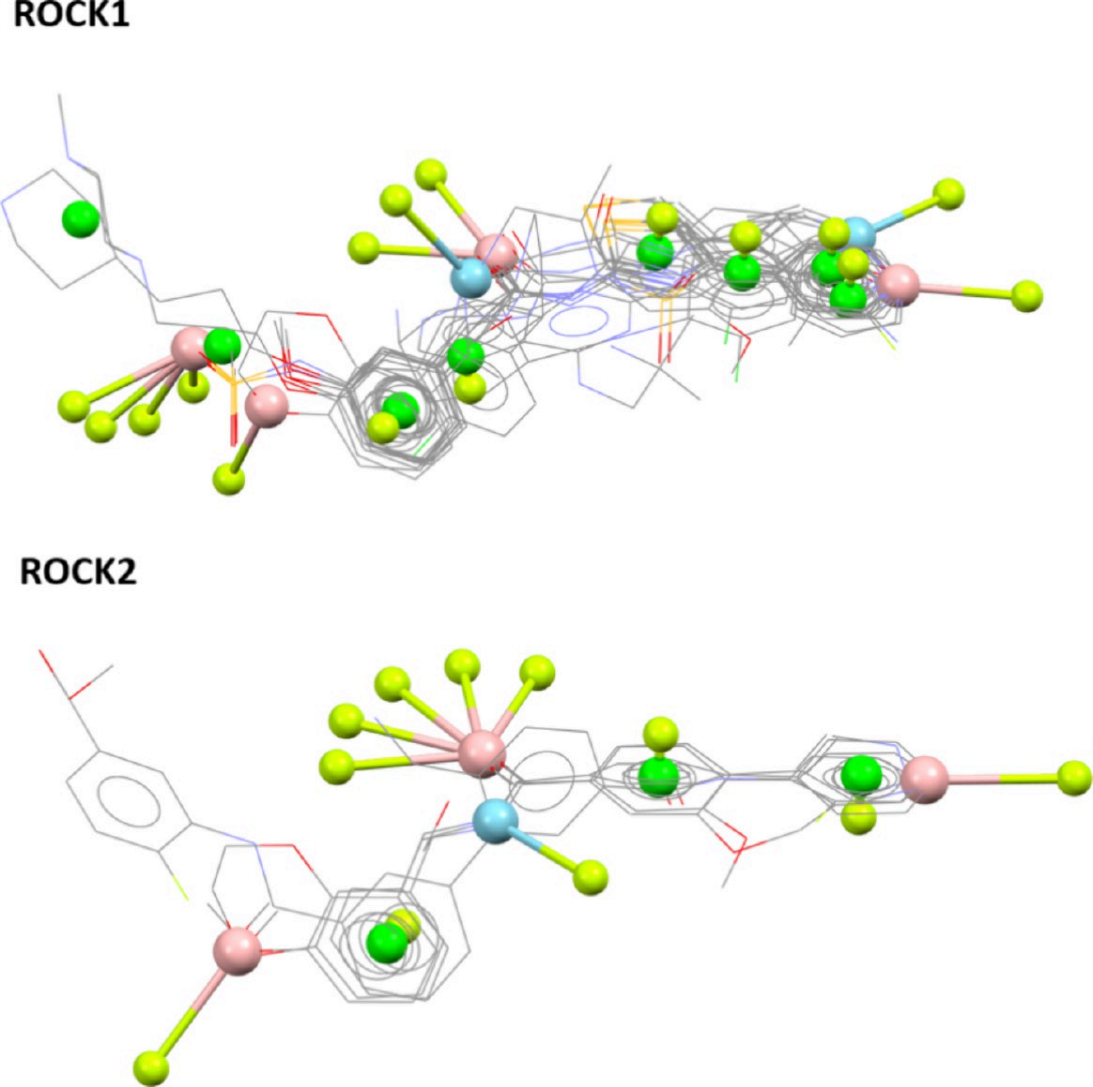
Pharmacophoric maps of ROCK1 and ROCK2 generated by Ligand Overlay Analysis.

The analysis of the resulting pharmacophoric maps allowed us to see that both maps are very similar despite the fact that they correspond to different ROCK isoforms. This can be explained by the fact of both isoforms being highly homologous, with 100% identity on the ATP binding site and a very close shape, as can be seen in Figure 4.

Analysis of the pharmacophoric maps allows the identification of interesting hotspots for the interaction with ROCK1/2 (Figure 6). The leading pharmacophoric point is the hydrogen bond acceptor related to the hydrogen bond interaction with the hinge region, which is a conserved interaction across all kinase inhibitors. For ROCK1 and ROCK2, this interaction is mediated by sp^2^ nitrogen in heterocyclic rings. For ROCK1, a hydrogen bond donor pharmacophoric point in the hinge binding region was also highlighted since some of the molecules can interact with the carbonyl oxygen of the main chain of Glu154 of ROCK1. Despite being absent from the obtained pharmacophore of ROCK2, probably due to fewer crystals available, this HBD point may also contribute to interactions with Glu170, given the structural homology between both sites and the lack of isoform selectivity of the inhibitors analyzed. In general, bidentate hinge binders provide mild improvement in inhibitory activity in both enzymes (Table 1).

One interesting pharmacophoric point includes a hydrogen bond acceptor at the linker position that corresponds to a hydrogen bond interaction with the side chain basic nitrogen of Lys105 of ROCK1 and Lys121 of ROCK2. Other interesting pharmacophoric points still include the aromatic ring centers related to the hinge binder motifs and the terminal aromatic rings that move out of plane, inserting into the affinity pocket and performing a cation-π interaction that is formed between an aromatic ring center and Lys105 of ROCK1 and Lys121 of ROCK2 residues. Other identified pharmacophoric points do not indicate any specific interactions.

On the other hand, the selected crystallographic ligands just cover a limited part of the entire chemical space of potent ROCK1/2 inhibitors, which raised the following question: would the identified pharmacophoric maps be relevant considering a representative fraction of the ROCKi chemical space? To answer this question, we decided to investigate the predictive capability of the solutions obtained from the Ligand Overlay algorithm by performing a ROC curve analysis.

### 2.4 Creation of the Virtual Screening Model for the Identification of ROCK Inhibitors

The active and inactive compounds for ROCK1 and 2 were selected from the ChEMBL database using a KNIME workflow that was developed to select representative fractions of the chemical space for active and inactive molecules. Since variability in assay conditions (enzyme type and concentration, substrate affinities and concentrations, buffer composition, detection systems) strongly influences IC_50_ values between works, inhibitory activity of substances from different sources must be compared using their dissociation constants (*K*_i_ or *K*_d_). Therefore, only compounds with known *K*_i_ or *K*_d_ values were selected and classified as active (*K*_i_ or *K*_d_ ≤ 100 nM) or inactive (*K*_i_ or *K*_d_ > 5000 nM). By applying filters for elimination of duplicates, compounds with unspecified stereochemistry, and a max MW of 550, we found 109 unique active compounds for ROCK1 and 163 active compounds for ROCK2, 375 inactive compounds for ROCK1 and 512 inactive compounds for ROCK2. To avoid bias in the pharmacophoric map validation, we performed a Hierarchical Clustering analysis on KNIME using a distance matrix calculation based on circular fingerprints to eliminate very similar compounds. The Hierarchical Cluster Assigner was set up based on a distance threshold of 0.5, resulting in 45 clusters for ROCK1 active compounds, 67 clusters for ROCK2 active compounds, 238 clusters for ROCK1 inactive compounds, and 346 clusters for ROCK2 inactive compounds (for the dendrogram visualization, see the Supporting Information, Figure S5). From each cluster, one compound was selected and prepared for analysis using the CCDC GOLD Ligand Prep node available in KNIME.

The prepared compounds were screened in the pharmacophoric maps of ROCK1/2 using the CSD Ligand Screener, and the ROC curves were traced. However, the AUC values obtained were poor: AUC = 0.68 for ROCK1 and AUC = 0.61 for ROCK2 (Figure 7).

**Figure 7:**
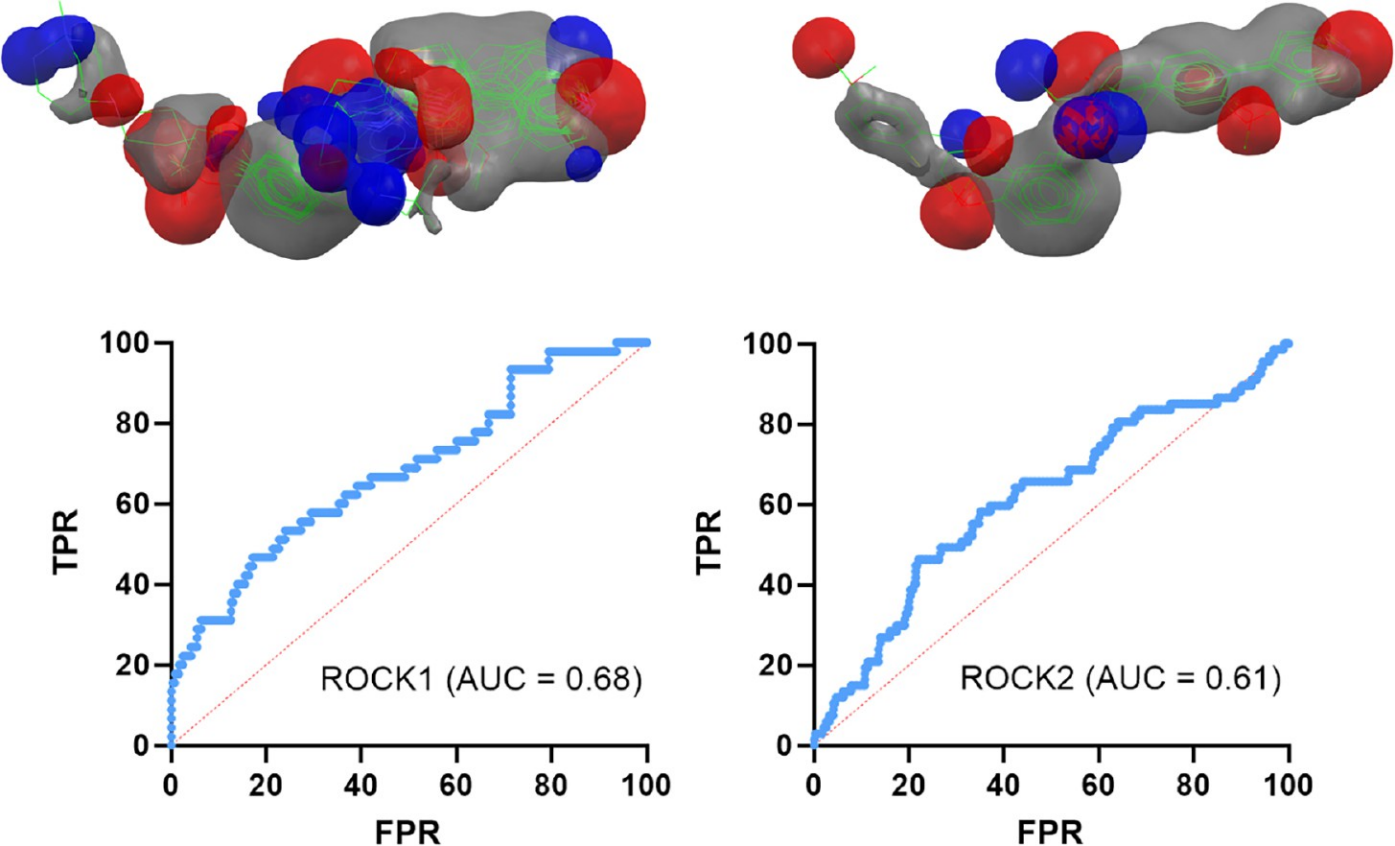
ROC curves obtained for ROCK1 and ROCK2 field-based virtual screening using the CSD Ligand Screener. TPR = true positive rate, FPR = false positive rate.

The poor AUC values can have two meanings: one is that lower chemical diversity of the cocrystallized ligands was not suitable enough for the determination of pharmacophoric maps for ROCK1/2, the other one is that the lack of protein-mediated steric effects was too detrimental to establish good ROC curves (AUC ≥ 0.75) for ROCK1/2. To answer our question, we decided to translate our calculated pharmacophoric maps into the protein environment and create a docking protocol to retrieve new ROC curves for ROCK1/2.

Using the GOLD’2022.3.0 program, we performed the docking analysis in ROCK1 (PDB: 6E9W) and ROCK2 (PDB: 7JNT). Initially, we performed redocking analysis of the cocrystallized ligands for the four scoring functions available on GOLD: ChemPLP, Chemscore, Goldscore, and ASP. Considering the RMSD values, the Goldscore function was selected. Next, we translated the pharmacophoric map information from Ligand Overlay to the docking analysis, using the Pharmacophore Constraint function available on GOLD, we added four pharmacophoric points, one hydrogen bond acceptor for the hinge region, one aromatic ring center in the hinge region, a hydrogen bond acceptor for the interaction with the catalytic Lys residue and an aromatic ring center for the affinity pocket formed between the catalytic Lys residue and glycine-rich loop (Figure 8). Additionally, we added a Protein Hydrogen Bond Constraint function available on GOLD, requiring a hydrogen bond interaction with the main chain of the methionine residue of the hinge region in both proteins (Met156 in ROCK1 and Met172 in ROCK2). The docking analysis of the active and inactive compounds was also performed with no constraint function in order to assess the significance of the methodology.

**Figure 8:**
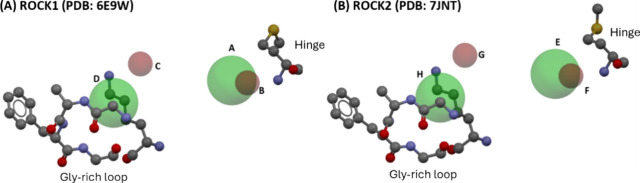
Pharmacophore constraint used on GOLD for ROCK1 (A) and ROCK2 (B). The pharmacophoric points are represented as green spheres for aromatic ring centers (A, D, E, and H) and red spheres for hydrogen bond acceptors (B, C, F, and G). Ball and stick models represent the hinge Met156/Met172, the catalytic K105/K121 and the glycine-rich loop of ROCK isoforms.

The newly generated ROC curves for our docking protocol are listed in Figure 9. Three conclusions can be made by the obtained AUC values; one is that using the Pharmacophore constraint functions available on GOLD, we truly improved our virtual screening protocol (^ROCK1^AUC = 0.80; ^ROCK2^AUC = 0.75) in comparison with the respective analysis without constraint (^ROCK1^AUC = 0.70; ^ROCK2^AUC = 0.61). Second, answering our question, the lack of protein-mediated steric effects was detrimental to establishing the ROC curves for ROCK1/2 using just the pharmacophoric maps with Ligand Overlay. This could be compensated for if it were possible to add different weights to the generated fitting points in the CSD Ligand Screener. Third, the identified pharmacophoric maps are relevant and representative of the ROCKi chemical space. Moreover, the pharmacophoric maps can help the future development of new ROCKi.

**Figure 9:**
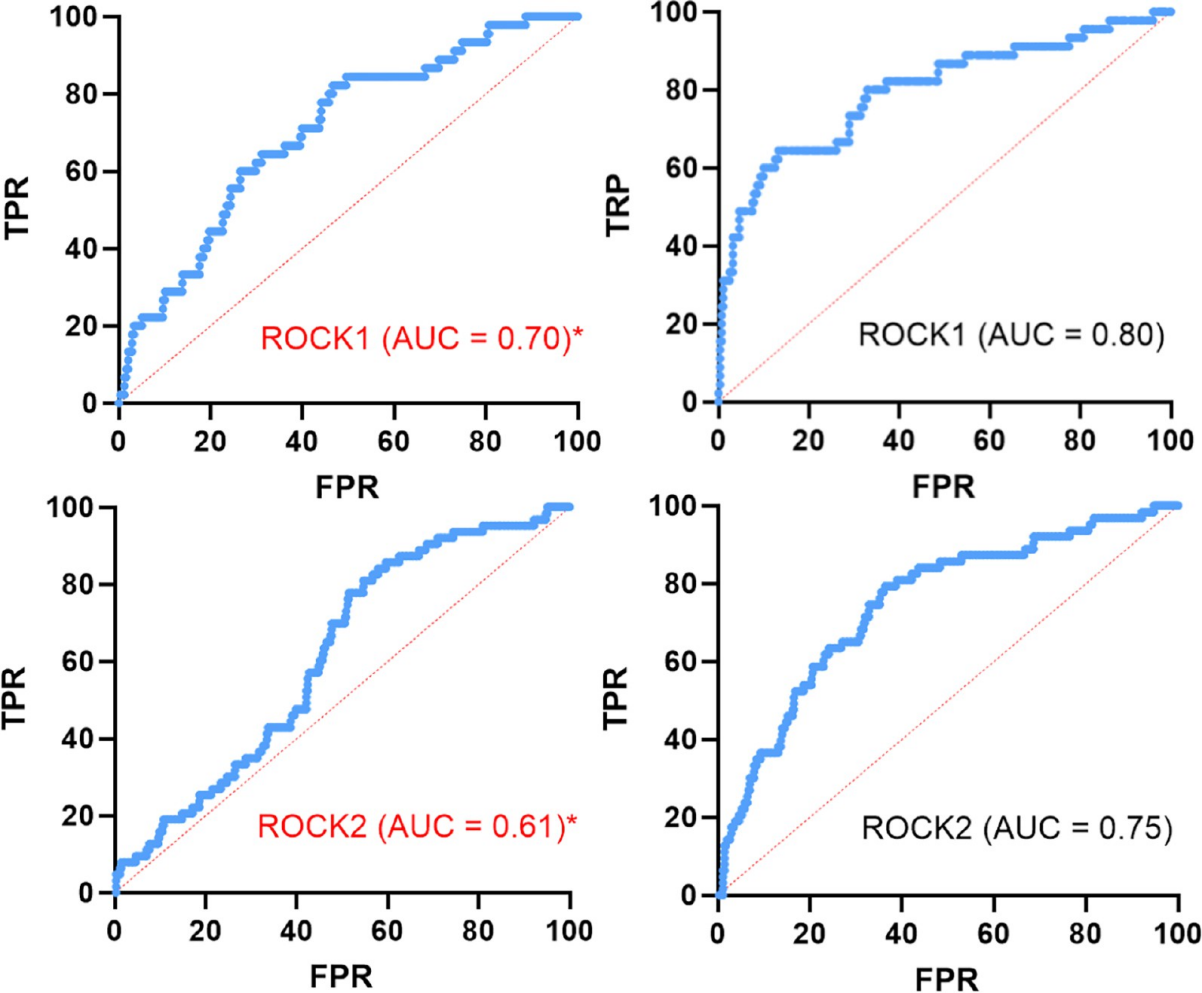
ROC curves obtained for ROCK1 and ROCK2 virtual screening using GOLD. *Analysis without constraint functions (red). TPR = true positive rate, FPR = false positive rate.

Analyzing the pharmacophoric maps along with the correspondent protein residues (Figure 8) it is clear that the hydrogen bond interaction with the main chain of the methionine residue (Met156 in ROCK1 and Met172 in ROCK2) is essential for molecular recognition of the ligands, which is not surprising since this kind of interaction is required for any Type I/II kinase inhibitor that binds in the ATP binding site. What was very surprising was that the interaction with the affinity pocket formed between the lysine residue (Lys105 in ROCK1 and Lys121 in ROCK2) and the glycine-rich loop is an important factor in gaining affinity, which brings new observations. The affinity pocket is out of plane in relation to the hinge region (Figure 8), meaning that a break of planarity is a necessary factor to reach these two pharmacophoric points. Beyond that, in general the affinity pocket is occupied by six-membered aromatic rings, performing a cation-π interaction with the catalytic lysine, and the aromatic rings are normally *meta*-substituted, which can be related to the size of the affinity pocket, *para*-substitution would probably result in steric repulsion promoted by the side chain of the phenylalanine residue (Phe87 in ROCK1 and Phe103 in ROCK2), moving the aromatic ring out of the affinity pocket.

### 2.5 Virtual Screening of Approved Drugs for the Identification of ROCK Inhibitors

After the virtual screening model creation and optimization based on ROC curves, we decided to experimentally validate it by identifying new ROCK inhibitors. The library of approved drugs was retrieved from the ChEMBL database, in which molecules with a determined IC_50_ and/or *K*_i_ for ROCK1 and/or ROCK2 were eliminated. Also, a previous filter to select drugs with hinge binder motifs was applied. The filter was based on drugs with nitrogenated heterocyclic aromatic rings with 5, 6, or 10 members. The resulting library with 500 approved drugs was docked, and then all drugs were visually analyzed, observing those that were able to reach at least the hinge region. Finally, a medicinal chemistry analysis was conducted using a structure-based rationale, focusing on the molecular fit within the ROCK binding site. This approach prioritized drugs that demonstrated favorable binding conformations with lower conformational energy, suggesting higher stability and affinity for ROCK. As a result, 15 drugs were selected for inhibition assays (Supporting Information, Table S3), of which 6 exhibited significant inhibitory activity at the tested concentrations (Table 2, Figure S1). Therefore, the efficiency of our virtual screening model, combined with medicinal chemistry analysis, was calculated as the proportion of active compounds, resulting in a 40% success rate (6 out of 15 compounds showing activity). From the identified ROCK inhibitors, the JAK inhibitors ruxolitinib (**36**) and baricitinib (**37**) were the most potent, presenting IC_50_ values in the low nanomolar range for both isoforms (Table 2, Figure S1). In addition, ponatinib (**38**) was also identified as a ROCK inhibitor in the nanomolar range, while tivozanib (**39**), nialamide (**40**), and tucatinib (**41**) presented IC_50_ values in the low micromolar range (Table 2, Figure S1). It is important to notice that tivozanib (**39**) showed 15-fold selectivity for ROCK2 over ROCK1.

**Table 2:**
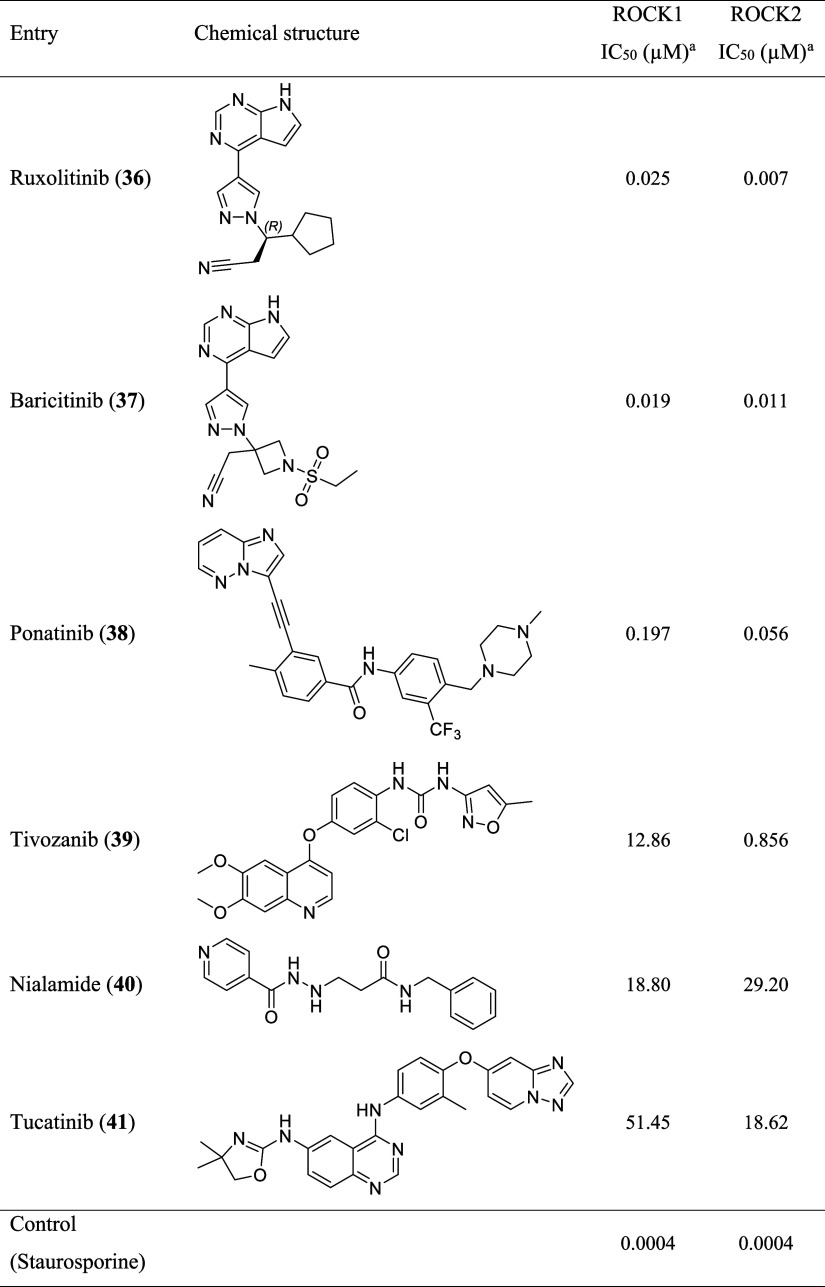
ROCK1/2 Inhibition Data

a Studies performed by Reaction Biology Corp., Malvern, PA (Study number: 20230203-UFRJ-CF-KP-RV01). Compounds were tested in 10-dose IC_50_ mode with 3-fold serial dilutions in duplicate using 1 μM ATP concentration.

Considering repurposing the identified ROCK inhibitors for neurodegenerative diseases, three critical factors were evaluated: (i) the primary target of the identified ROCK inhibitors should have implications in neurodegenerative conditions and potentially interact with the RhoA/ROCK signaling pathway; (ii) the inhibitors should exhibit high potency for ROCK inhibition selectivity across the kinome, minimizing off-target effects; (iii) the identified inhibitors should be capable of crossing the blood-brain barrier (BBB).

Considering that ruxolitinib (**36**) and baricitinib (**37**) are the most potent ROCK inhibitors identified, exhibit notable selectivity within the kinome, and act as JAK inhibitors with anti-inflammatory activity, they emerge as promising candidates for the treatment of neurodegenerative diseases. Additionally, both drugs have been previously reported to cross the blood-brain barrier (BBB),^49–51^ further supporting their repositioning potential. In fact, JAK inhibitors have already been highlighted as promising candidates for neurodegenerative conditions,^52,53^ with baricitinib (**37**) being specifically proposed for Alzheimer’s disease treatment.^54,55^ It is also worth noting that while ruxolitinib’s (**36**) ROCK inhibitory activity had been previously reported in terms of percentage inhibition at 1 μM,^56^ IC_50_ values for both drugs were unavailable in the literature. Ruxolitinib (**36**) was selected for further evaluation based on these criteria.

Ruxolitinib (**36**) interacts with ROCK1 and ROCK2 through bidentate hydrogen bonds with glutamate and methionine residues in the hinge region (Figure 10). The pyrazole ring acts as a linker, directing the cyclopentane ring toward the affinity pocket. Notably, ruxolitinib (**36**) does not interact with the catalytic lysine. Differences were observed in the alkyl nitrile group’s positioning: it faces the solvent in ROCK1 (Figure 10A) but inserts into a lipophilic subpocket formed by Leu221 and Ala231 in ROCK2 (Figure 10B).

**Figure 10:**
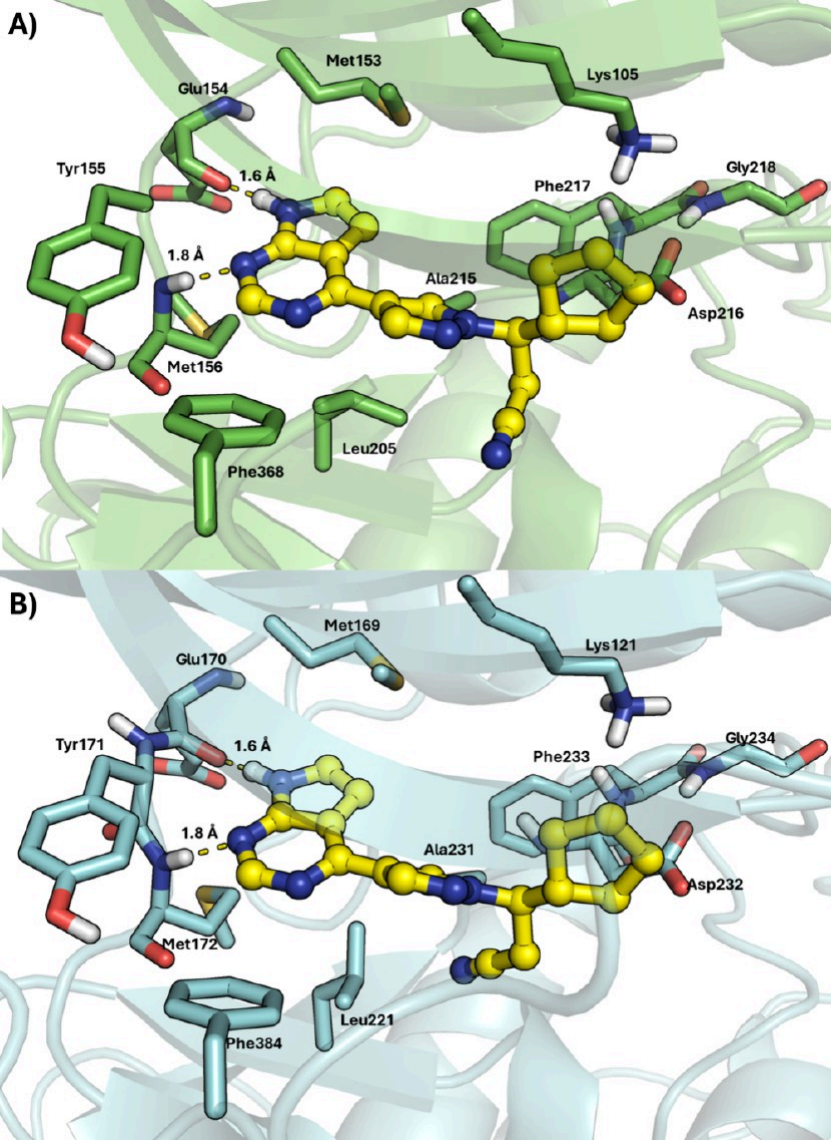
Calculated interaction mode of ruxolitinib (**36**) in (a) ROCK1 (PDB: 6E9W) and (b) ROCK2 (PDB: 7JNT). Ruxolitinib (**36**) carbon atoms are shown in yellow.

Ruxolitinib (**36**) was also evaluated against other kinases closely related to ROCK within the AGC family (Table 3, Figure S2). Ruxolitinib’s (**36**) selectivity for ROCK1 and ROCK2 over other kinases in the AGC family suggests a favorable off-target profile within this kinase group. According to Table 3, its IC_50_ values for MLCK/MYLK, PAK1, PKAcβ, PKG1α, and PKCα are significantly higher than those for ROCK1 and ROCK2, indicating less potent inhibition of these kinases. Specifically, the IC_50_ for MLCK/MYLK is 792 nM, for PAK1 is greater than 10,000 nM, and for PKAcβ is 1314 nM. This selective inhibition profile in the AGC family underscores the potential of ruxolitinib (**36**) to effectively target neurodegenerative pathways by modulating ROCK/JAK activities while minimizing off-target effects.

**Table 3:**
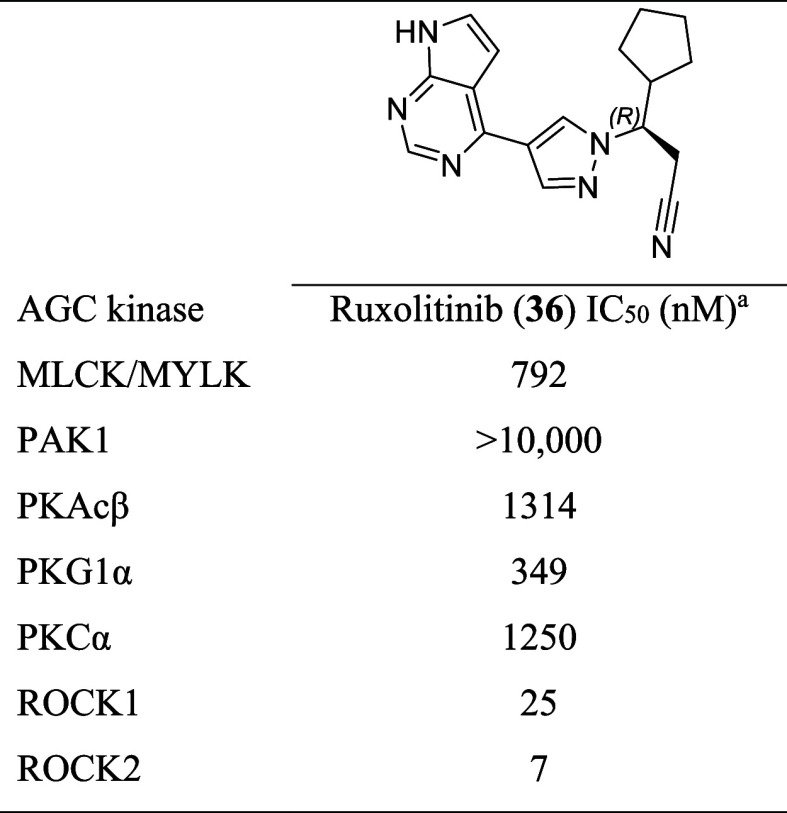
Selectivity of Ruxolitinib (**36**) for ROCK1 and ROCK2 in the AGC Kinase Family

a Studies performed by Reaction Biology Corp., Malvern, PA (Study number: 20230203-UFRJ-CF-KP-RV01; 20240122-UFRJ-CF-KP). Compounds were tested in 10-dose IC_50_ mode with 3-fold serial dilutions in duplicate using 1 μM ATP concentration.

However, as a pan-JAK inhibitor, ruxolitinib is associated with hematologic side effects (e.g., neutropenia and thrombocytopenia) due to JAK1/2 inhibition. While its ROCK selectivity may offer therapeutic benefits in neurodegenerative diseases, concurrent JAK inhibition could pose safety risks. To mitigate this, future analogs could prioritize structural modifications that enhance ROCK selectivity over JAK isoforms or employ lower doses to minimize JAK-mediated toxicity. Additionally, monitoring hematologic parameters in preclinical models would be essential to evaluating the risk-benefit balance of repurposing JAK inhibitors for neurodegenerative applications.

Next, we conducted molecular dynamics simulations to understand Ruxolitinib’s (**36**) stability with ROCK1 and ROCK2.

### 2.6 Molecular Dynamics Simulations with Ruxolitinib (**36**)

To understand the similar potency of ruxolitinib (**36**) in inhibiting ROCK1 and ROCK2, the stability of the ROCK1- and ROCK2-ruxolitinib (**36**) complexes was assessed by analyzing the root-mean-square deviation (RMSD) of the protein heavy atoms and ruxolitinib (**36**) atoms over three independent 100 ns simulation trajectories. For ROCK1, the RMSD fluctuated around 3.0 Å, indicating that the protein maintained its structural integrity throughout the simulations (Figure 11a,b). Similarly, Ruxolitinib (**36**) exhibited an RMSD fluctuating around 2.5 Å (Figure 11c,d), remaining stable over the course of the experiment, which suggests a strong binding interaction.

**Figure 11:**
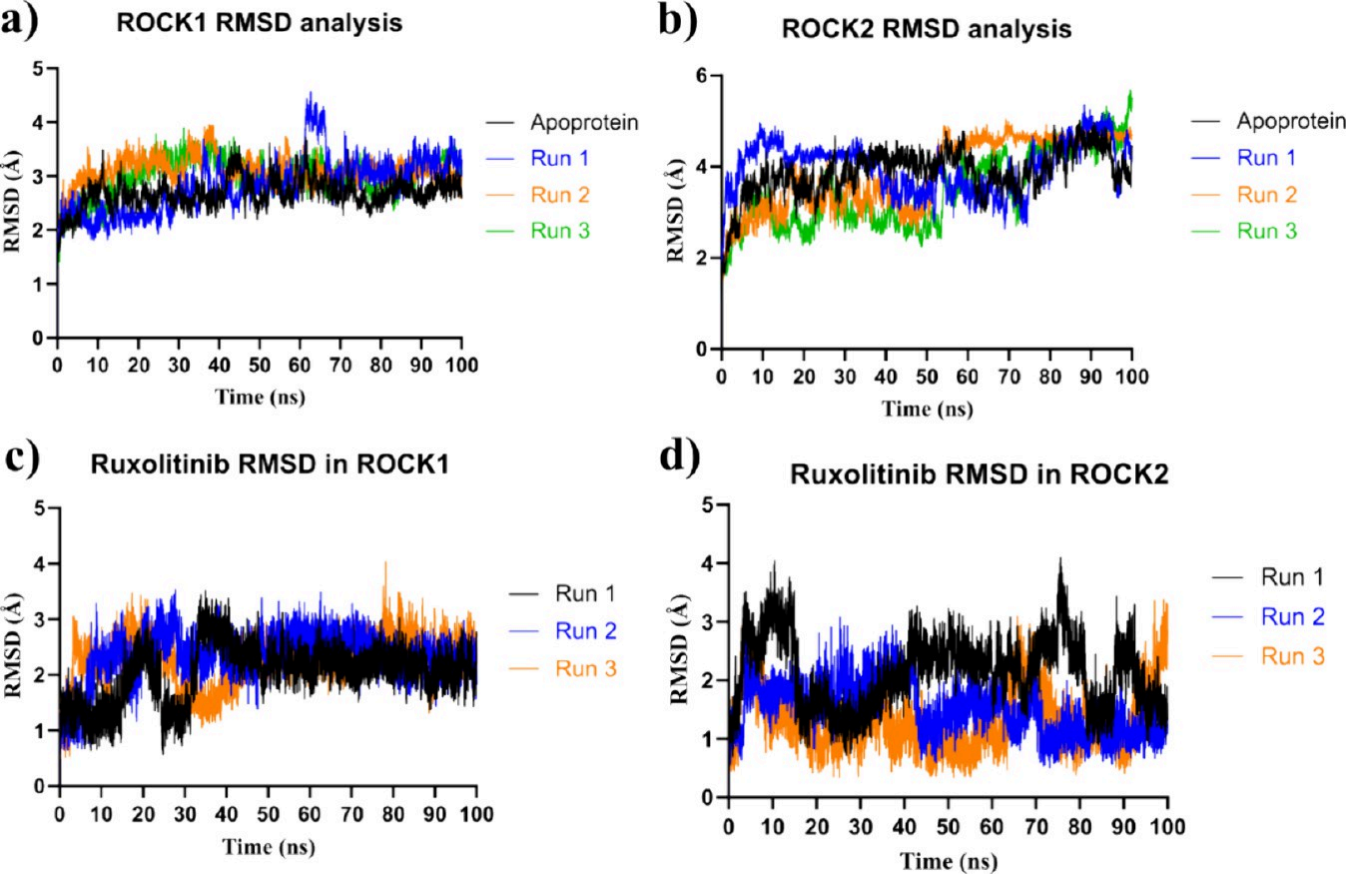
RMSD analysis of ROCK1 and ROCK2 protein–ligand complexes during molecular dynamics simulations over a 100 ns simulation time across three independent runs. (a) RMSD of the ROCK1 heavy atoms. (b) RMSD of the ROCK2 heavy atoms. (c) RMSD of the Ruxolitinib (**36**) atoms within the ROCK1 complex. (d) RMSD of the ruxolitinib (**36**) atoms within the ROCK2 complex.

To further investigate the flexibility of the protein–ligand complexes, the root-mean-square fluctuation (RMSF) of each residue of ROCK1 and ROCK2 was calculated (Figure 12). Most residues in both proteins showed low fluctuations (<3.0 Å), indicating a relatively rigid protein structure (Figure 12a,b). More importantly, comparisons of the simulations for the apoproteins and the different runs with the ligands showed low fluctuations, indicating no induced fit based on the presence of the ligand.

**Figure 12:**
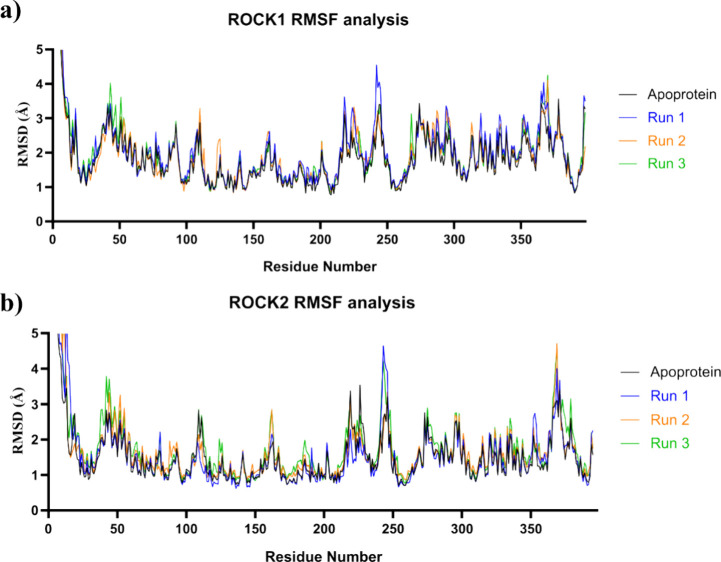
RMSF analysis of ROCK1 and ROCK2 protein–ligand complexes during molecular dynamics simulations over the course of 100 ns simulations. (a) RMSF of each residue in the ROCK1. (b) RMSF of each residue in the ROCK2.

Hydrogen bonds between ruxolitinib (**36**) and the hinge region (Glu154 and Met156 of ROCK1, and Glu170 and Met172 of ROCK2) were analyzed throughout the simulations to understand their contribution to binding stability. On average, hydrogen bonds in the hinge region were consistently maintained during the different simulation runs (Figure 13). The persistence of these hydrogen bonds was further analyzed by measuring the distance between the hydrogen and heteroatom, which showed values lower than 3.0 Å over the course of the experiment, consistent with hydrogen bonding (Figure 13). Thus, by analyzing the molecular dynamics simulations of ruxolitinib (**36**) in ROCK1 and ROCK2, we observed a similar profile, which could explain the similar activity of this drug in both kinases.

**Figure 13:**
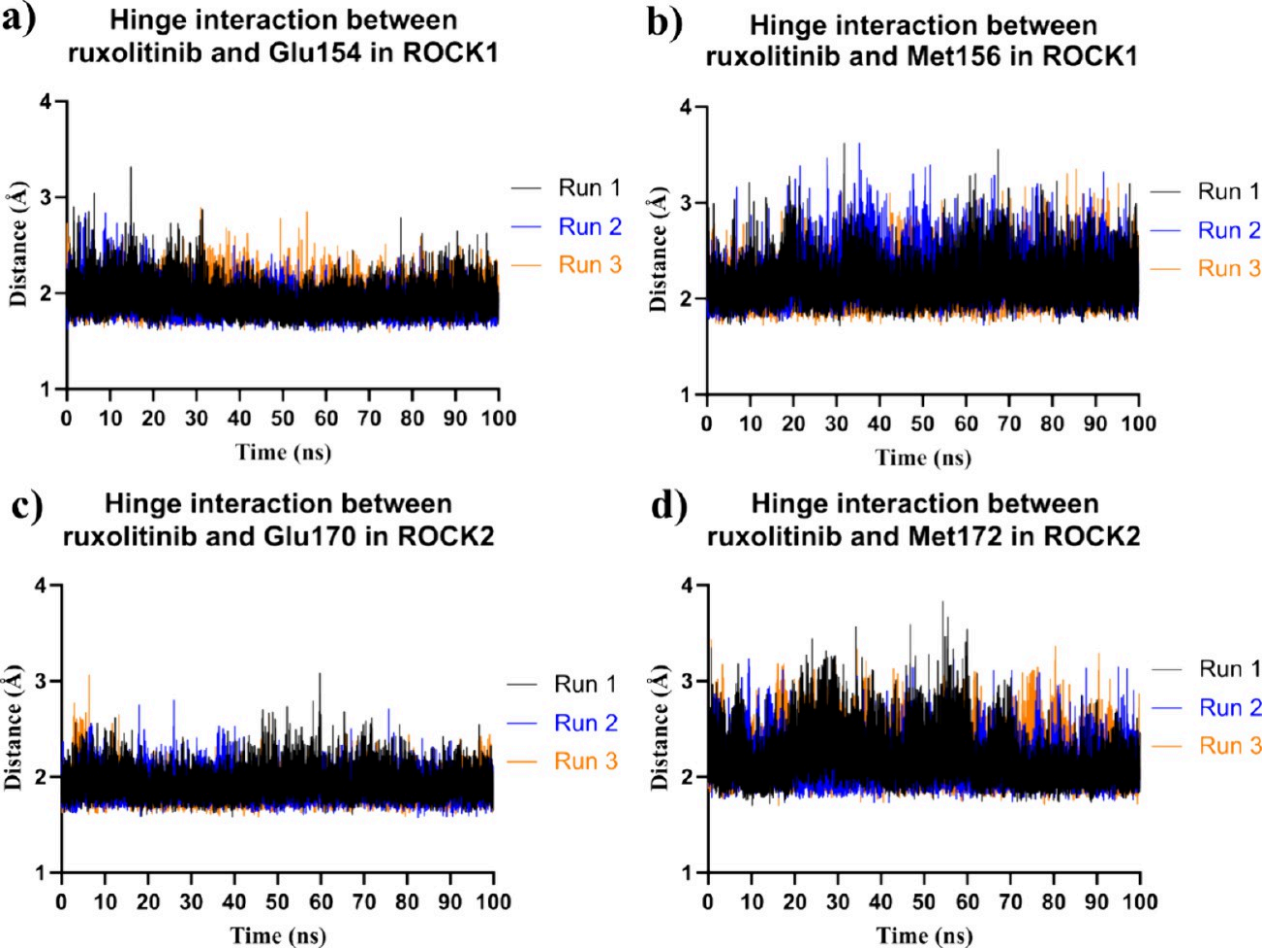
Analysis of hydrogen bond interactions between ruxolitinib (**36**) and the hinge regions of ROCK1 and ROCK2 during molecular dynamics simulations. (a) Time-dependent analysis of the hydrogen bonds between Ruxolitinib (**36**) and the hinge region residue Glu154 of ROCK1. (b) Time-dependent analysis of the hydrogen bonds between Ruxolitinib (**36**) and the hinge region residues Met156 of ROCK1. (c) Time-dependent analysis of the hydrogen bonds between ruxolitinib (**36**) and the hinge region residues Glu170 of ROCK2. (d) Time-dependent analysis of the hydrogen bonds between Ruxolitinib (**36**) and the hinge region residues Met172 of ROCK2.

To further support these findings, thermodynamic binding free energy calculations were performed using the MM/GBSA approach based on the generated simulation trajectories. For ROCK1, the average Δ*G* was −30.76 ± 0.30 kcal/mol, while for ROCK2, it was −29.59 ± 1.21 kcal/mol (see Supporting Information and Table S4). These values are in good agreement with the experimental inhibitory activities (ROCK1 IC_50_ = 0.025 μM; ROCK2 IC_50_ = 0.007 μM), reinforcing the conclusion that ruxolitinib (36) exhibits comparable affinity and potency toward both kinase isoforms.

### 2.7 Biological Validation of Ruxolitinib Effects on Neural Cells In Vitro

The pharmacological evaluation of ruxolitinib (**36**) underscores its potential as a therapeutic agent, particularly in the context of neurodegenerative diseases. To evaluate the effects of ruxolitinib (**36**) on neural cells in vitro, distinct neural cell lines and primary cultures were exposed to different concentrations of the compound. The cell viability assays demonstrated that ruxolitinib (**36**), at concentrations ranging from 0.1 to 5 μM, did not adversely affect the viability of astrocytic and neuronal cell lines (Figure 14a,b). Further, the cell morphology was not affected by ruxolitinib (**36**). Astrocytes exhibit a complex GFAP staining network characteristic of this cell type. Treatment with ruxolitinib (**36**) did not impact intermediate filament organization and process extension (Figure 14c). To evaluate the effect of ruxolitinib (**36**) on neuroinflammation, we treated murine primary astrocytes with LPS (lipopolysaccharide), a well-known inducer of neuroinflammation. As shown in Figure 14c, LPS increases the levels of the complement component C3, a marker associated with neuroinflammation and astrocyte toxicity.^57^ Interestingly, ruxolitinib decreased C3 immunostaining in LPS-treated astrocyte cultures (Figure 14c). Our results are in good agreement with previous evaluations of ruxolitinib (**36**).^58,59^

**Figure 14:**
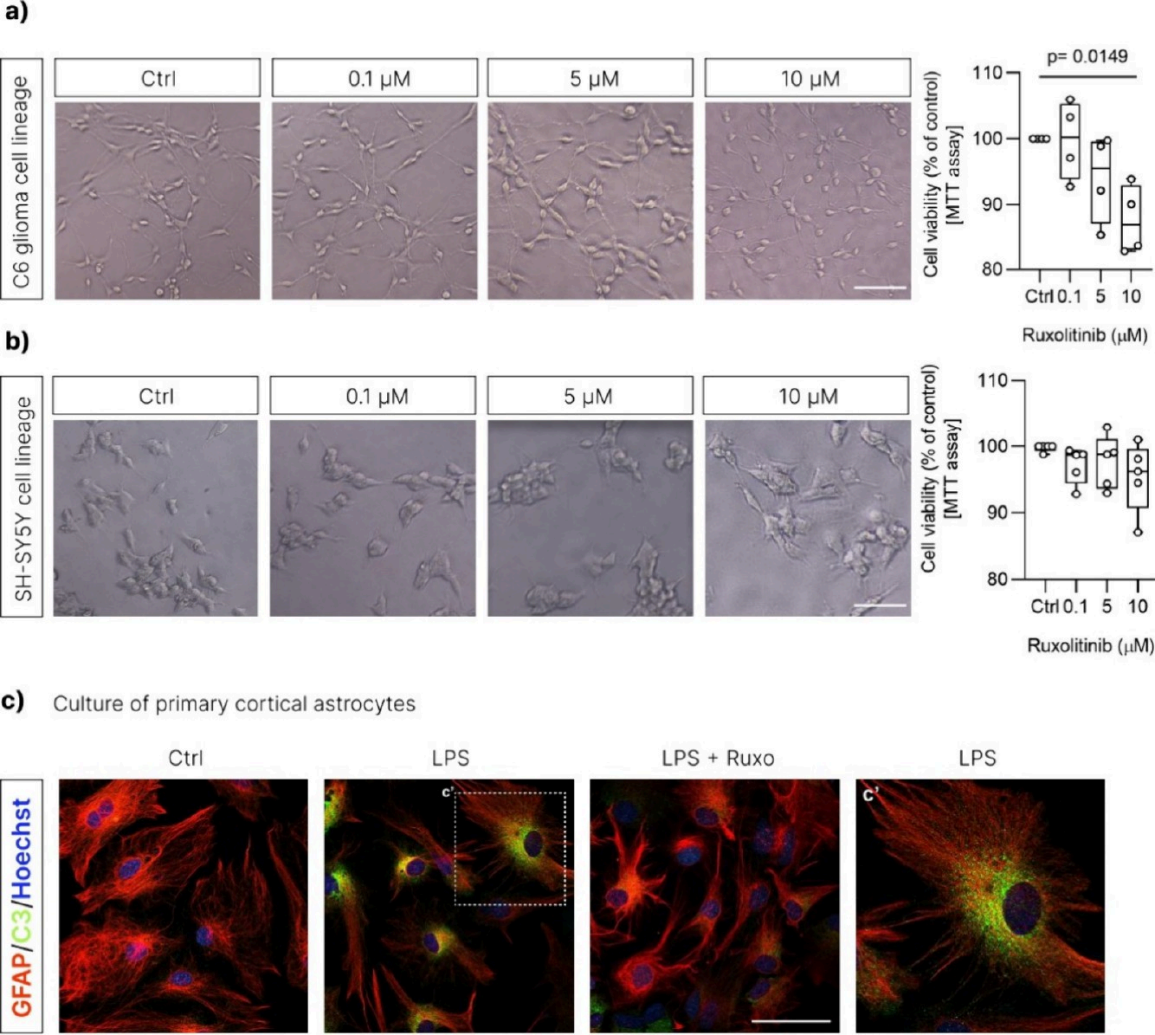
Effects of ruxolitinib on neuronal and astrocyte cell lines and primary cortical astrocytes. C6 glioma (a) and SH-SY5Y (b) cell lines were exposed to different concentrations of ruxolitinib ranging from 0.1 to 10 μM for 24 h. No significant impact on the viability of those cells were observed between the concentrations of 0.1 and 5 μM­(graphics, a, b). Panel shows bright-field microscopy images of C6 glioma and SH-SY5Y cell lines. (c) Astrocyte primary cultures were exposed to LPS only or to LPS followed by treatment with 1 μM of ruxolitinib for 24 h. After, cells were immunolabeled for GFAP and C3. Whereas LPS increased the levels of C3 in GFAP positive cells; ruxolitinib reversed this event. Ctrl: control astrocytes; LPS: LPS-treated astrocytes; LPS + ruxolitinib: astrocytes exposed to LPS and ruxolitinib. One-way ANOVA with Dunnett’s multiple comparisons. Data are shown as the mean ± SD (*n* = 4–5 biological repeats). Scale bars: 50 μm.

Taken together, those results are promising, as they indicate: 1. a favorable safety profile for ruxolitinib (**36**) at these concentrations, which is essential for its potential therapeutic use; 2. an anti-inflammatory effect of ruxolitinib (**36**), which is particularly relevant in the context of neurodegenerative diseases, where inflammation often plays a crucial role in disease progression. These findings warrant further investigation through in vitro and in vivo studies to fully elucidate ruxolitinib’s (**36**) therapeutic potential and its underlying mechanisms of action. Ultimately, these studies could pave the way for the development of new effective treatments for neurodegenerative diseases.

## 3 Conclusions

This study conducted an in-depth analysis of the structural elements responsible for inhibiting ROCK1/2 at low nanomolar concentrations. Although greater chemical diversity may exist beyond our criteria, we encountered a limited number of reported inhibitors with known dissociation constants, which ultimately restricted the execution of a larger screening algorithm. Nevertheless, we identified the main pharmacophoric features necessary for the rational design of new inhibitors and emphasized the critical interactions with the two isoforms of ROCK. Specifically, we highlighted the importance of interaction within a subcavity orthogonal to the ATP binding site, referred to as the ROCK affinity pocket, located between the glycine-rich loop and the catalytic lysine residue. These observations prompted us to pursue a virtual screening campaign focused on drug repurposing for neurodegeneration. Our virtual screening studies yielded a 40% efficiency protocol, leading to the identification of ruxolitinib (**36**) and baricitinib (**37**) as highly potent ROCK1/ROCK2 inhibitors, with IC_50_ values in the low nanomolar range, making them promising candidates for repurposing. To further characterize these drugs, ruxolitinib (**36**) was selected for subsequent steps, demonstrating high selectivity for ROCK1 and ROCK2 within the AGC kinase family and highlighting its JAK/ROCK multitarget profile rather than a promiscuous pan-kinase inhibitor.

Molecular dynamics (MD) simulations revealed the stability of ruxolitinib’s (**36**) interactions with ROCK1/ROCK2, opening new avenues for the design of JAK/ROCK multitarget-directed ligands for the treatment of neurodegenerative disorders. Additionally, in vitro pharmacological assays demonstrated that ruxolitinib (**36**) does not affect the viability of astrocytic and neuronal cell lines at concentrations ranging from 0.1 to 5 μM and may possess anti-inflammatory and antitoxicity properties.

In conclusion, this study provides a solid foundation for the future development of ruxolitinib-based treatments for neurodegenerative diseases. Further validation through *in vitro* and *in vivo* experiments should elucidate its therapeutic potential and mechanisms of action.

## 4 Methodology

### 4.1 Chemical Space Analysis of ROCK Inhibitors

Chemical spaces of ROCK1/2 ligands were analyzed using the KNIME platform.^31^ Smiles strings were transformed to ECFP_6 (2048 bits) chemical fingerprints using RDKit.^32,60^ Z-score and Minimum-Maximum normalization, PCA and MDS dimensionality reduction, mean silhouette coefficient calculation, and clustering algorithms (hierarchical clustering, *k*-means, and *k*-medoids) were performed using KNIME nodes. Chemical space depiction was obtained using a 2*D*/3D Scatterplot KNIME node provided by the Erlwood cheminformatics extension.

### 4.2 Selection of Active and Inactive ROCK1/2 Inhibitors

The active and inactive compounds for ROCK1 and 2 were selected from the ChEMBL database using a KNIME workflow (See Supporting Information). First, we used the REST API of ChEMBLdb to extract the data set of ligands for the biological targets ChEMBL ID (ROCK1: CHEMBL3231; ROCK2: CHEMBL2973) using *K*_i_ and *K*_d_ data.^61^ After applying filters, the ligands data set was split into active (pchembl_value ≥ 7) and inactive compounds (standard_value ≥ 5000), resulting in 4 data sets, active and inactive for ROCK1/active and inactive for ROCK2. Later, salt counterions were removed, and then molecules with unspecified stereocenters and compounds with a MW ≥ 550 were removed as well. Circular fingerprints were determined for the remaining molecules, and the distance matrix calculation was performed using Tanimoto as a distance function. Next, hierarchical clustering analysis was performed using a cluster assignment based on a distance threshold of 0.5. From each resulting cluster, one compound was selected and prepared as a high-quality 3D structure through the CCDC GOLD Ligand Prep^61^ node. Finally, the ionization and tautomeric states were manually adjusted.

### 4.3 Ligand Overlay Analysis and Field-Based Virtual Screening

The ligands reported in Table 3 were protonated and extracted from their respective crystal structures. Using the Ligand Overlay^48^ wizard available from Hermes of the CCDC package, we performed a pharmacophoric map calculation for ROCK1 and ROCK2. The analysis was performed without conformer generation. The calculation was performed using the default setting without constraints. The selected solutions were the top-ranked solutions for each run. The field-based virtual screening^47^ analysis was performed using the previously selected solutions from the Ligand Overlay analysis for fitting points generation. Next, the respective active and inactive compounds for ROCK1 and ROCK2 were screened over the generated fitting points with a maximum number of conformers for each ligand set up to 200. Based on the obtained screening scores, a ROC curve was traced for each target. Pharmacophore models were visualized using a CCDC Hermes.

### 4.4 Docking Analysis and Virtual Screening

For the docking analysis, the crystal structure of ROCK1–6E9W^37^ and the crystal structure of ROCK2–7JNT^19^ were selected. From the cocrystallized ligands, a 10 Å radius was used for the binding site selection. A redocking analysis of the cocrystallized ligands for each crystal was performed to select the best scoring function available on GOLD’2022.3.0 based on RMSD values. The selected scoring function was Goldscore for ROCK1 and ROCK2. For the docking analysis of the active and inactive compounds, the pharmacophore constraint functions were set up as described below with the x, y, and z coordinates, the radius in Å, and the constraint weight:

- ROCK1 (6E9W): point 1 (H-bond acceptor)–*x*: 50.3; *y*: 98.8; *z*: 23.7; radius–0.5; constraint weight = 10. Point 2 (H-bond acceptor)–*x*: 50.6; *y*: 105.4; *z*: 30.1; radius–0.7; constraint weight = 5. Point 3 (aromatic ring center)–*x*: 50.5; *y*: 99.6; *z*: 24.9; radius–2; constraint weight = 5. Point 4 (aromatic ring center)–*x*: 48.7.5; *y*: 104.7; *z*: 34.2; radius–2; constraint weight = 5.
- ROCK2 (7JNT): point 1 (H-bond acceptor)–*x*: −5.8; *y*: −5.0; *z*: −34.8; radius–0.5; constraint weight = 10. Point 2 (H-bond acceptor)–*x*: −2.3; *y*: 2.3; *z*: −30.5; radius–0.7; constraint weight = 5. Point 3 (aromatic ring center)–*x*: −5.4; *y*: −3.9; *z*: −34.2; radius–2; constraint weight = 5. Point 4 (aromatic ring center)–*x*: −1.6; *y*: 5.8; *z*: −33.1; radius–2; constraint weight = 5.

In addition, the Protein HBond constraint function was used to require a hydrogen bond interaction with MET156:H of ROCK1 and MET172:H of ROCK2 using a constraint weight of 10 and a minimum H-bond geometry weight of 0.005. In order to evaluate the constraint methodology, a docking analysis was also performed without constraints. The ROC curves were traced by using the goldscore.fitness data. Ligand-protein interaction diagrams were generated using PyMOL (v. 1.4).

### 4.5 Molecular Dynamics and MM/GBSA Calculations

MD simulations were performed in FlarePro+ using the Open MM package (V6.1, Cresset, Litlington, Cambridgeshire, UK; https://www.cresset-group.com/software/flare/). We performed MD simulations on the docked complex of ruxolitinib (**36**) with the ROCK1 (PDB ID: 6E9W)^37^ and ROCK2 (PDB ID: 7JNT).^19^ Ruxolitinib (**36**) was minimized using the Open 2.0.0 force field and protein with the AMBER force field. MD simulation is performed with default settings with a normal calculation method; the simulation length is 100 ns. The transferable intermolecular potential with 3 points (TIP3P) water model was used to describe the solvent water; the AM1-BCC charge method was applied. By default, the protein–ligand complex was the first production run. After the completion, the simulation trajectory was analyzed for the RMSD (root-mean-square deviation) plot and protein–ligand contacts. MM/GBSA calculations were performed by using generated trajectories in FlarePro+. The first 20% of the trajectories were discarded. The interval between trajectory frames was set to 1, and the solvent model used was implicit GBn2.

### 4.6 Materials

Lipopolysaccharides from *Salmonella enterica* serotype typhimurium (LPS, L6143 - Sigma-Aldrich); DMEM/F12 (Dulbecco’s minimum essential medium and nutrient mixture F-12; Invitrogen); FBS (Fetal bovine serum; Invitrogen); MTT (Sigma-Aldrich); Normal goat serum (Sigma-Aldrich); ruxolitinib, baricitinib, tucatinib, ponatinib, tivozanib, dasabuvir, cilostazole, raltegravir, erdafitinib, doravirine, sertindole (Ambeed, USA); nialamide, picotamide, rimegepant, brexpiprazole (TargetMol, USA).

### 4.7 High-Performance Liquid Chromatography

Analytical HPLC was performed for compound purity determinations using a Shimadzu LC-20AT with a Thermo Scientific Hypersil BDS C18 column (4.6 × 250 mm) and a Shimadzu SPD-20AV detector. The solvent system used for the HPLC analyses was acetonitrile/water at 7:3. The isocratic HPLC mode was used, and the flow rate was 1.0 mL/min. The purity of the compounds was higher than 95%. Figure S3 shows the chromatogram of ruxolitinib (**36**).

### 4.8 Kinase Inhibition Biochemical Assays

The HotSpot Kinase Assay was used for compound screening on protein kinases, as already reported.^62^ The assay was performed in a base reaction buffer containing 20 mM Hepes (pH 7.5), 10 mM MgCl_2_, 1 mM EGTA, 0.01% Brij-35, 0.02 mg/mL BSA, 0.1 mM Na_3_VO_4_, 2 mM DTT, and 1% DMSO, with the required cofactors added individually to each kinase reaction. The substrate was prepared in freshly made base reaction buffer, and cofactors were added accordingly. The indicated kinase was then delivered into the substrate solution and gently mixed. Test compounds, dissolved in 100% DMSO, were added to the kinase reaction mixture using acoustic technology (Echo550; nanoliter range), followed by incubation for 20 min at room temperature. The reaction was initiated by adding ^33^P-ATP (1 μM) and incubating for 2 h at room temperature. Kinase activity was detected using the P81 filter-binding method.

### 4.9 Ruxolitinib Preparation and Dosages

Ruxolitinib (Ambeed, USA) was dissolved in dimethyl sulfoxide (DMSO), and the stock solution (5 mM) and working solution (1 mM) were kept at −20 °C and only thawed to room temperature before use. To SH-SY5Y and C6 cell cultures, a range of ruxolitinib concentrations (0–10 μM; 0.1% DMSO) was used by adding the appropriate amount of the working solution into the culture media, whereas to primary cortical astrocytes, ruxolitinib was prepared at 1 μM; 0.1% DMSO solution.

### 4.10 Animals

Newborn (postnatal days 0–3) C57BL/6N mice (*Mus musculus*) were obtained from the Federal University of Rio de Janeiro (UFRJ), Rio de Janeiro, Brazil. Animals were housed under a 12 h light-dark cycle with free access to water and food. All experimental procedures were carried out in accordance with the approval of the Animal Use Ethics Committee of the Federal University of Rio de Janeiro (CEUA-UFRJ, approval protocols A23/21-006/18 and 122/22). Experiments were performed according to Brazilian Guidelines on Care and Use of Animals for Scientific and Teaching Purposes (DBCA).

### 4.11 Cell Culture

C6 cells (BCRJ Code: 0057, ATCC: CCL-107) and SH-SY5Y cells (BCRJ Code: 0223, ATCC: CRL-2266) were maintained at 37 °C in a 5% CO_2_ humidified incubator. Prior to the experiments, SH-SY5Y cells were cultured in DMEM/F12 plus 10% FBS (Fetal bovine serum, Invitrogene). For further experiments, cells were divided into four groups, depending on the treatment conditions: cells were treated with DMEM (Ctrl group), or with different concentrations of ruxolitinib, 0.1, 5, and 10 μM (ruxolitinib group) for 24 h. C6 cells were cultured in DMEM/F12 with 2.5% FBS (Invitrogen) and divided into four groups: the Ctrl group and ruxolitinib groups (0.1, 5, and 10 μM). The cells were incubated for 24 h under these conditions.

### 4.12 Astrocyte Cultures

Primary cortical astrocyte cultures were prepared from brains of P0–3 C57BL/6 mice as described by Matias et al.^63^ The meninges of the brain were removed to cortex desiccation in Gey’s buffer, and the cerebral cortex was maintained in DMEM/F12, supplemented with 10% FBS. Cultures were incubated at 37 °C in a humidified 5% CO_2_, 95% air chamber for approximately 7 DIV until confluence, with medium exchange every 2 days. After confluence, cultures were separated into three experimental groups: control (0.1% DMSO), LPS (10 μg/mL; 0.1% DMSO) and ruxolitinib treatment (1 μM; 0.1% DMSO) + LPS (10 μg/mL) in DMEM/F12 with 10% FBS for 24 h. After 24 h, cultures were washed and fixed.

### 4.13 Cell Viability and Morphology Analysis

C6 and SH-SY5Y cells were plated in a 96-well plate with 1 × 10^4^ cells per well. Cell morphology was observed using an EVOS phase-contrast microscope (Invitrogen, ThermoFisher Scientific, USA), and viability was evaluated using a 3-[4,5-dimethylthiazol-2-yl]-2,5-diphenyltetrazolium bromide (MTT) reduction assay. After 24 h of treatment, cells were incubated with 0.5 mg/mL MTT dye for 3 h at 37 °C and 5% CO_2_. This assay evaluates the labeling activity of mitochondrial dehydrogenases such as succinate dehydrogenase by reducing MTT (yellow) to a purple formazan salt. The culture media were removed, and the crystals were dissolved in 150 μL DMSO. The intensity of formazan was measured using a GloMax spectrophotometer (Promega) at a wavelength of 560 nm. This assay was performed according to the protocol described by Mosmann (1983).^64^

### 4.14 Immunocytochemistry

Astrocyte cultures were fixed with 4% PFA in phosphate-buffered saline (PBS, pH 7.4) for 15 min, and nonspecific sites were blocked with 3% BSA, 5% normal goat serum, and 0.2% Triton X-100 diluted in PBS for 1 h, before incubation with the rabbit anti-GFAP (1:1,000, DAKO Cytomation, Cat. Z0334) and the rat anti-C3 (1:100, Abcam, Cat. ab11862) at 4 °C overnight. Subsequently, the cells were thoroughly washed with PBS and incubated with secondary antibodies at room temperature for 2 h. Secondary antibodies were Alexa Fluor Plus 488 antirabbit plus (1:800, Thermo Fisher Scientific) and Alexa Fluor 546 antirat (1:1000, Thermo Fisher Scientific). Nuclei were counterstained with Hoechst 33342 (Sigma-Aldrich), and cells were observed with a Zeiss Celldiscoverer 7.

### 4.15 Statistical Analysis

*In vitro* data were presented as mean ± SD, analyzed by One-way analysis of variance (ANOVA) followed by Dunnett’s or Tukey’s multiple comparisons test. Data are shown as the mean ± SD (*n* = 3–5 biological repeats). Results were considered significant when the p-value was less than 0.05. All analyses and graphs were performed with GraphPad Prism 9 for MacOs (v 9.1.1) statistical software.

## Supplementary Material



## Data Availability

The SMILES representations of the active and inactive compounds used for model validation, along with their ChEMBL activity values, are provided as supporting files in CSV format. The molecular models of human ROCK1 (PDB: 6E9W) and ROCK2 (PDB: 7JNT), including the docking pose of ruxolitinib (**36**) used as input for MD simulations, are available in MOL2 format. All files can be found at https://github.com/pedrosenamp/ROCK_virtual_screening.

## Notes

Hierarchical clustering, *k*-means, and *k*-medoids methods to analyze ROCK1/2 ligands, with PCA (Table S1) and MDS (Table S2); ROCK1/2 inhibition data for all compounds selected in the VS campaign (Table S3); IC_50_ curves for ROCK1 and ROCK2 inhibition by ruxolitinib, baricitinib, tivozanib, tucatinib, ponatinib, and nialamide, as well as the control compound staurosporine (Figure S1); IC_50_ curves for ruxolitinib against PAK1, MLCKMYLK, PKG1a, PKCa, PKAcb, ROCK1, and ROCK2 (Figure S2); chromatogram of ruxolitinib (Figure S3); overlay of selected ROCK1 and ROCK2 crystal structures (Figure S4); MM/GBSA data obtained from ruxolitinib’s MD trajectories in ROCK1 and ROCK2 (Table S4); and dendrograms for the hierarchical clustering of active and inactive compounds for ROCK1 and ROCK2 based on circular fingerprint similarities (Figure S5). (PDF)

## #

C.A.M.F. Deceased on May 8, 2024.

## Abbreviations

Aβ: amyloid-β
AD: Alzheimer’s disease
ATP: adenosine triphosphate
AUC: area under the curve
CCDC: Cambridge Crystallographic Datacenter
CSD: Cambridge Structure Database
JAK: Janus kinase
LPS: lipopolysaccharide
MDS: multidimensional scaling
MLCK/MYLK: myosin light chain kinase
PAK1: p21-activated kinase 1
PCA: principal component analysis
PKAcβ: protein kinase A catalytic subunit beta
PKCα: protein kinase C alpha
PKG1α: protein kinase G 1 alpha
RMSD: root-mean-square deviation
RMSF: root-mean-square fluctuation
ROC: receiver operating characteristic
ROCK: Rho-associated protein kinases
ROCKi: ROCK inhibitor.

## References

[1] Matsui T., Amano M., Yamamoto T., Chihara K., Nakafuku M., Ito M., Nakano T., Okawa K., Iwamatsu A., Kaibuchi K. (1996). Rho-Associated
Kinase, a Novel Serine/Threonine Kinase, as a Putative Target for
Small GTP Binding Protein Rho. EMBO J..

[2] Riento K., Ridley A. J. (2003). ROCKs: Multifunctional Kinases in Cell Behaviour. Nat. Rev. Mol. Cell Biol..

[3] Nakagawa O., Fujisawa K., Ishizaki T., Saito Y., Nakao K., Narumiya S. (1996). ROCK-I and ROCK-II,
Two Isoforms of Rho-Associated
Coiled-Coil Forming Protein Serine/Threonine Kinase in Mice. FEBS Lett..

[4] Tolomeu H. V., Fraga C. A. M. (2022). The Outcomes of Small-Molecule Kinase
Inhibitors and
the Role of ROCK2 as a Molecular Target for the Treatment of Alzheimer’s
Disease. CNS Neurol Disord Drug Targets.

[5] Hu Y., Ren R., Zhang Y., Huang Y., Cui H., Ma C., Qiu W., Wang H., Cui P., Chen H., Wang G. (2019). Rho-associated
Coiled-coil Kinase 1 Activation Mediates Amyloid Precursor Protein
Site-specific Ser655 Phosphorylation and Triggers Amyloid Pathology. Aging Cell.

[6] Herskowitz J. H., Feng Y., Mattheyses A. L., Hales C. M., Higginbotham L. A., Duong D. M., Montine T. J., Troncoso J. C., Thambisetty M., Seyfried N. T., Levey A. I., Lah J. J. (2013). Pharmacologic Inhibition
of ROCK2 Suppresses Amyloid- Production in an Alzheimer’s Disease
Mouse Model. J. Neurosci..

[7] Henderson B. W., Gentry E. G., Rush T., Troncoso J. C., Thambisetty M., Montine T. J., Herskowitz J. H. (2016). Rho-Associated
Protein Kinase 1 (ROCK1)
Is Increased in Alzheimer’s Disease and ROCK1 Depletion Reduces
Amyloid-β Levels in Brain. J. Neurochem.

[8] Gao Y., Yan Y., Fang Q., Zhang N., Kumar G., Zhang J., Song L.-J., Yu J., Zhao L., Zhang H.-T., Ma C.-G. (2019). The Rho Kinase Inhibitor
Fasudil Attenuates Aβ1–42-Induced
Apoptosis via the ASK1/JNK Signal Pathway in Primary Cultures of Hippocampal
Neurons. Metab Brain Dis.

[9] Hamano T., Shirafuji N., Yen S.-H., Yoshida H., Kanaan N. M., Hayashi K., Ikawa M., Yamamura O., Fujita Y., Kuriyama M., Nakamoto Y. (2020). Rho-Kinase ROCK Inhibitors Reduce
Oligomeric Tau Protein. Neurobiol Aging.

[10] Shinozaki Y., Danjo Y., Koizumi S. (2019). Microglial
ROCK Is Essential for
Chronic Methylmercury-induced Neurodegeneration. J. Neurochem.

[11] Rodriguez-Perez A. I., Borrajo A., Rodriguez-Pallares J., Guerra M. J., Labandeira-Garcia J. L. (2015). Interaction
between NADPH-Oxidase and Rho-Kinase in Angiotensin II-Induced Microglial
Activation. Glia.

[12] Hensel N., Rademacher S., Claus P. (2015). Chatting with the Neighbors: Crosstalk
between Rho-Kinase (ROCK) and Other Signaling Pathways for Treatment
of Neurological Disorders. Front Neurosci.

[13] Forgione N., Fehlings M. G. (2014). Rho-ROCK Inhibition
in the Treatment of Spinal Cord
Injury. World Neurosurg.

[14] Kubo T. (2008). The Therapeutic
Effects of Rho-ROCK Inhibitors on CNS Disorders. Ther Clin Risk Manag.

[15] Tan H.-B., Zhong Y.-S., Cheng Y., Shen X. (2011). Rho/ROCK Pathway and
Neural Regeneration: A Potential Therapeutic Target for Central Nervous
System and Optic Nerve Damage. Int. J. Ophthalmol..

[16] Labandeira-Garcia J.
L., Rodríguez-Perez A. I., Villar-Cheda B., Borrajo A., Dominguez-Meijide A., Guerra M. J. (2015). Rho Kinase and Dopaminergic
Degeneration. Neuroscientist.

[17] Hoy S. M. (2018). Netarsudil
Ophthalmic Solution 0.02%: First Global Approval. Drugs.

[18] Blair H. A. (2021). Correction
to: Belumosudil: First Approval. Drugs.

[19] Hu Z., Wang C., Sitkoff D., Cheadle N. L., Xu S., Muckelbauer J. K., Adam L. P., Wexler R. R., Quan M. L. (2020). Identification
of 5H-Chromeno­[3,4-c]­Pyridine and 6H-Isochromeno­[3,4-c]­Pyridine Derivatives
as Potent and Selective Dual ROCK Inhibitors. Bioorg. Med. Chem. Lett..

[20] Garnock-Jones K. P. (2014). Ripasudil:
First Global Approval. Drugs.

[21] SONE T. (1996). Development
of Fasudil Hydrochloride (Eril): A New Protein Kinase Inhibitor. *Journal of Synthetic Organic*. Chemistry.

[22] Sturdivant J. M., Royalty S. M., Lin C.-W., Moore L. A., Yingling J. D., Laethem C. L., Sherman B., Heintzelman G. R., Kopczynski C. C., deLong M. A. (2016). Discovery of the ROCK Inhibitor Netarsudil
for the Treatment of Open-Angle Glaucoma. Bioorg.
Med. Chem. Lett..

[23] Blair H. A. (2021). Belumosudil:
First Approval. Drugs.

[24] Jacobs M., Hayakawa K., Swenson L., Bellon S., Fleming M., Taslimi P., Doran J. (2006). The Structure
of Dimeric ROCK I Reveals
the Mechanism for Ligand Selectivity. J. Biol.
Chem..

[25] Ashburn T. T., Thor K. B. (2004). Drug Repositioning: Identifying and Developing New
Uses for Existing Drugs. Nat. Rev. Drug Discov.

[26] Li J., Zheng S., Chen B., Butte A. J., Swamidass S. J., Lu Z. (2016). A Survey of Current
Trends in Computational Drug Repositioning. Brief Bioinform.

[27] Pushpakom S., Iorio F., Eyers P. A., Escott K. J., Hopper S., Wells A., Doig A., Guilliams T., Latimer J., McNamee C., Norris A., Sanseau P., Cavalla D., Pirmohamed M. (2019). Drug Repurposing: Progress, Challenges
and Recommendations. Nat. Rev. Drug Discov.

[28] Yu M. J. (2013). Druggable
Chemical Space and Enumerative Combinatorics. J. Cheminform.

[29] Giuliani A. (2017). The Application
of Principal Component Analysis to Drug Discovery and Biomedical Data. Drug Discov Today.

[30] Agrafiotis D. K., Rassokhin D. N., Lobanov V. S. (2001). Multidimensional Scaling and Visualization
of Large Molecular Similarity Tables. J. Comput.
Chem..

[31] Berthold, M. R. ; Cebron, N. ; Dill, F. ; Gabriel, T. R. ; Kötter, T. ; Meinl, T. ; Ohl, P. ; Sieb, C. ; Thiel, K. ; Wiswedel, B. KNIME: The Konstanz Information Miner. In Data Analysis, Machine Learning and Applications; 2008; Springer: Berlin, Heidelberg; pp. 319–326.

[32] Rogers D., Hahn M. (2010). Extended-Connectivity Fingerprints. J. Chem.
Inf Model.

[33] Gaulton A., Hersey A., Nowotka M., Bento A. P., Chambers J., Mendez D., Mutowo P., Atkinson F., Bellis L. J., Cibrián-Uhalte E., Davies M., Dedman N., Karlsson A., Magariños M. P., Overington J. P., Papadatos G., Smit I., Leach A. R. (2017). The ChEMBL Database
in 2017. Nucleic Acids Res..

[34] Martin E., Cao E. (2015). Euclidean Chemical
Spaces from Molecular Fingerprints: Hamming Distance
and Hempel’s Ravens. J. Comput. Aided
Mol. Des.

[35] Struyf A., Hubert M., Rousseeuw P. (1996). Clustering
in an Object-Oriented
Environment. J. Stat. Softw..

[36] Peters J.-U. (2013). Polypharmacology–Foe
or Friend?. J. Med. Chem..

[37] Hobson A. D., Judge R. A., Aguirre A. L., Brown B. S., Cui Y., Ding P., Dominguez E., DiGiammarino E., Egan D. A., Freiberg G. M., Gopalakrishnan S. M., Harris C. M., Honore M. P., Kage K. L., Kapecki N. J., Ling C., Ma J., Mack H., Mamo M., Maurus S., McRae B., Moore N. S., Mueller B. K., Mueller R., Namovic M. T., Patel K., Pratt S. D., Putman C. B., Queeney K. L., Sarris K. K., Schaffter L. M., Stoll V., Vasudevan A., Wang L., Wang L., Wirthl W., Yach K. (2018). Identification of Selective Dual
ROCK1 and ROCK2 Inhibitors Using Structure-Based Drug Design. J. Med. Chem..

[38] Ginn J. D., Bosanac T., Chen R., Cywin C., Hickey E., Kashem M., Kerr S., Kugler S., Li X., Prokopowicz A., Schlyer S., Smith J. D., Turner M. R., Wu F., Young E. R. R. (2010). Substituted 2H-Isoquinolin-1-Ones as Potent Rho-Kinase
Inhibitors: Part 2, Optimization for Blood Pressure Reduction in Spontaneously
Hypertensive Rats. Bioorg. Med. Chem. Lett..

[39] Pireddu R., Forinash K. D., Sun N. N., Martin M. P., Sung S.-S., Alexander B., Zhu J.-Y., Guida W. C., Schönbrunn E., Sebti S. M., Lawrence N. J. (2012). Pyridylthiazole-Based Ureas as Inhibitors
of Rho Associated Protein Kinases (ROCK1 and 2). Medchemcomm.

[40] Patel R. A., Forinash K. D., Pireddu R., Sun Y., Sun N., Martin M. P., Schönbrunn E., Lawrence N. J., Sebti S. M. (2012). RKI-1447
Is a Potent Inhibitor of the Rho-Associated ROCK Kinases with Anti-Invasive
and Antitumor Activities in Breast Cancer. Cancer
Res..

[41] Shaw D., Hollingworth G., Soldermann N., Sprague E., Schuler W., Vangrevelinghe E., Duggan N., Thomas M., Kosaka T., Waters N., van Eis M. J. (2014). Novel ROCK Inhibitors for the Treatment
of Pulmonary Arterial Hypertension. Bioorg.
Med. Chem. Lett..

[42] Green J., Cao J., Bandarage U. K., Gao H., Court J., Marhefka C., Jacobs M., Taslimi P., Newsome D., Nakayama T., Shah S., Rodems S. (2015). Design, Synthesis,
and Structure–Activity
Relationships of Pyridine-Based Rho Kinase (ROCK) Inhibitors. J. Med. Chem..

[43] Gao H., Marhefka C., Jacobs M. D., Cao J., Bandarage U. K., Green J. (2018). ROCK Inhibitors 2. Improving Potency,
Selectivity and Solubility
through the Application of Rationally Designed Solubilizing Groups. Bioorg. Med. Chem. Lett..

[44] Bandarage U. K., Cao J., Come J. H., Court J. J., Gao H., Jacobs M. D., Marhefka C., Nanthakumar S., Green J. (2018). ROCK Inhibitors 3:
Design, Synthesis and Structure-Activity Relationships of 7-Azaindole-Based
Rho Kinase (ROCK) Inhibitors. Bioorg. Med. Chem.
Lett..

[45] Hu Z., Wang C., Glunz P. W., Li J., Cheadle N. L., Chen A. Y., Chen X.-Q., Myers J. E., Guarino V. R., Rose A., Sack J. S., Sitkoff D., Taylor D. S., Xu S., Yan C., Zhang H., Zhang L., Hennan J., Adam L. P., Wexler R. R., Quan M. L. (2020). Discovery of a Phenylpyrazole
Amide ROCK Inhibitor as a Tool Molecule for in Vivo Studies. Bioorg. Med. Chem. Lett..

[46] Boland S., Bourin A., Alen J., Geraets J., Schroeders P., Castermans K., Kindt N., Boumans N., Panitti L., Fransen S., Vanormelingen J., Stassen J. M., Leysen D., Defert O. (2015). Design, Synthesis,
and Biological Evaluation of Novel,
Highly Active Soft ROCK Inhibitors. J. Med.
Chem..

[47] Giangreco I., Mukhopadhyay A., Cole J. C. (2021). Validation of a
Field-Based Ligand
Screener Using a Novel Benchmarking Data Set for Assessing 3D-Based
Virtual Screening Methods. J. Chem. Inf Model.

[48] Giangreco I., Olsson T. S. G., Cole J. C., Packer M. J. (2014). Assessment of a
Cambridge Structural Database-Driven Overlay Program. J. Chem. Inf Model.

[49] Ebadi M., Wasko J., Weisdorf D. J., Gordon P. M., Rashidi A. (2019). Ruxolitinib
Combined with Chemotherapy Can Eradicate Chemorefractory Central Nervous
System Acute Lymphoblastic Leukaemia. Br. J.
Hamaetol..

[50] Haile W. B., Gavegnano C., Tao S., Jiang Y., Schinazi R. F., Tyor W. R. (2016). The Janus Kinase Inhibitor Ruxolitinib Reduces HIV
Replication in Human Macrophages and Ameliorates HIV Encephalitis
in a Murine Model. Neurobiol Dis.

[51] Goker
Bagca B., Ozates N. P., Biray Avci C. (2023). Ruxolitinib
Enhances Cytotoxic and Apoptotic Effects of Temozolomide on Glioblastoma
Cells by Regulating WNT Signaling Pathway-Related Genes. Medical Oncology.

[52] Rusek M., Smith J., El-Khatib K., Aikins K., Czuczwar S. J., Pluta R. (2023). The Role of the JAK/STAT
Signaling Pathway in the Pathogenesis of
Alzheimer’s Disease: New Potential Treatment Target. Int. J. Mol. Sci..

[53] Qin H., Buckley J. A., Li X., Liu Y., Fox T. H., Meares G. P., Yu H., Yan Z., Harms A. S., Li Y., Standaert D. G., Benveniste E. N. (2016). Inhibition of the JAK/STAT Pathway
Protects Against α-Synuclein-Induced Neuroinflammation and Dopaminergic
Neurodegeneration. J. Neurosci..

[54] Hindam M. O., Ahmed L. A., Sayed E. L., Khattab M., Sallam N. A. (2024). Repositioning
of Baricitinib for Management of Memory Impairment in Ovariectomized/D-Galactose
Treated Rats: A Potential Role of JAK2/STAT3-PI3K/AKT/MTOR Signaling
Pathway. Life Sci..

[55] Faquetti M. L., Slappendel L., Bigonne H., Grisoni F., Schneider P., Aichinger G., Schneider G., Sturla S. J., Burden A. M. (2024). Baricitinib
and Tofacitinib Off-target Profile, with a Focus on Alzheimer’s
Disease. Alzheimer’s Dementia: Transl.
Res. Clin. Intervent..

[56] Zhou T., Georgeon S., Moser R., Moore D. J., Caflisch A., Hantschel O. (2014). Specificity and Mechanism-of-Action of the JAK2 Tyrosine
Kinase Inhibitors Ruxolitinib and SAR302503 (TG101348). Leukemia.

[57] Diniz L. P., Morgado J., Bergamo Araujo A. P., da Silva Antônio L. M., Mota-Araujo H. P., de Sena Murteira Pinheiro P., Sagrillo F. S., Cesar G. V., Ferreira S. T., Figueiredo C. P., Manssour Fraga C. A., Gomes F. C. A. (2024). Histone Deacetylase Inhibition Mitigates
Cognitive Deficits and Astrocyte Dysfunction Induced by Amyloid-β
(Aβ) Oligomers. Br. J. Pharmacol..

[58] Rudolph J., Heine A., Quast T., Kolanus W., Trebicka J., Brossart P., Wolf D. (2016). The JAK Inhibitor
Ruxolitinib Impairs
Dendritic Cell Migration via Off-Target Inhibition of ROCK. Leukemia.

[59] Min J., Zheng H., Xia H., Tian X., Liang M., Zhang J., Huang W. (2024). Ruxolitinib
Attenuates Microglial
Inflammatory Response by Inhibiting NF-ΚB/MAPK Signaling Pathway. Eur. J. Pharmacol..

[60] RDKit: Open-Source Cheminformatics. http://www.rdkit.org.

[61] 03_ChEMBL_Bioactivity_Search. https://hub.knime.com/-/spaces/-/latest/-tGSG1nPdGg4cW7Qp/https://hub.knime.com/-/spaces/-/latest/tGSG1nPdGg4cW7Qp/.

[62] Anastassiadis T., Deacon S. W., Devarajan K., Ma H., Peterson J. R. (2011). Comprehensive
Assay of Kinase Catalytic Activity Reveals Features of Kinase Inhibitor
Selectivity. Nat. Biotechnol..

[63] Matias I., Diniz L. P., Damico I. V., Araujo A. P. B., Neves L. D. S., Vargas G., Leite R. E. P., Suemoto C. K., Nitrini R., Jacob-Filho W., Grinberg L. T., Hol E. M., Middeldorp J., Gomes F. C. A. (2022). Loss of Lamin-B1 and Defective Nuclear Morphology Are
Hallmarks of Astrocyte Senescence in Vitro and in the Aging Human
Hippocampus. Aging Cell.

[64] Mosmann T. (1983). Rapid Colorimetric
Assay for Cellular Growth and Survival: Application to Proliferation
and Cytotoxicity Assays. J. Immunol Methods.

