# Supplementary material for: Drug Repurposing by Virtual Screening: Identification of New Already Approved ROCK Inhibitors as Promising Drugs to Target Neurodegeneration
Source: ACS Omega. 2025 Jun 26;10(26):28446–65. doi: 10.1021/acsomega.5c04340 (PMC12242638; doi:10.1021/acsomega.5c04340)
Supplement: Supplementary file 1 [file ao5c04340_si_001.pdf]

Supporting Informmation

Drug Repurposing by Virtual Screening: identification of new already approved ROCK inhibitors as promising drugs to target neurodegeneration

Lucas Silva Franco,[1, 2] Daniel Alencar Rodrigues,[2, 3] Gabriela Joras Baumart,[4] Bárbara da Silva Mascarenhas de Jesus,[1, 2, 5] Flávia Carvalho Alcantara Gomes,[4] Lídia Moreira Lima, [1, 2, 5] Carlos Alberto Manssour Fraga,[†, 1, 2, 5] Pedro de Sena Murteira Pinheiro,[, 1, 2, 5]*

1- Laboratório de Avaliação e Síntese de Substâncias Bioativas (LASSBio), Instituto de Ciências Biomédicas, Universidade Federal do Rio de Janeiro, 21941-902, Rio de Janeiro, Brazil.

2- Instituto Nacional de Ciência e Tecnologia de Fármacos e Medicamentos (INCT-INOFAR), Instituto de Ciências Biomédicas, Universidade Federal do Rio de Janeiro, 21941-902, Rio de Janeiro, Brazil.

3- School of Pharmacy and Biomolecular Sciences (PBS), Royal College of Surgeons in Ireland, 1st Floor Ardilaun House Block B 111 St Stephen's Green, Dublin 2, Ireland.

4- Laboratório de Neurobiologia Celular, Programa de Pós-Graduação em Ciências Morfológicas, Instituto de Ciências Biomédicas, Universidade Federal do Rio de Janeiro, 21941-902, Rio de Janeiro, Brazil.

5- Programa de Pós-Graduação em Farmacologia e Química Medicinal, Instituto de Ciências Biomédicas, Universidade Federal do Rio de Janeiro, 21941-902, Rio de Janeiro, Brazil.

† - *C.A.M.F. Deceased on May 8, 2024*

*To whom correspondence should be addressed. E-mail: pedro.pinheiro@icb.ufrj.br

Table S1. PCA based maximum mean silhouette coefficient for ROCK1/2 ligands employing hierarchical clustering, k-means, and k-medoids methods.

Data set	Clustering method	Maximum mean silhouette score (number of clusters, k)
ROCK1 (166 molecules)	Hierarchical clustering	0.91 (k = 3)
	K-means	0.56 (k = 4)
	K-medoids	0.56 (k = 3)
ROCK2 (218 molecules)	Hierarchical clustering	0.92 (k = 3)
	K-means	0.89 (k = 3)
	K-medoids	0.91 (k = 3)

Table S2. MDS based maximum mean silhouette coefficient for ROCK1/2 ligands employing hierarchical clustering, k-means, and k-medoids methods.

Data set	Clustering method	Maximum mean silhouette score (number of clusters, k)
ROCK1 (159 molecules)	Hierarchical clustering	0.09 (k = 3)
	K-means	0.44 (k = 4)
	K-medoids	0.24 (k = 3)
ROCK2 (199 molecules)	Hierarchical clustering	0.19 (k = 9)
	K-means	0.47 (k = 6)
	K-medoids	0.29 (k = 3)

Table S3. ROCK1/2 inhibition data.

Entry	Structure	IC_{50} ROCK1 (µM)	IC_{50} ROCK2 (µM)
Picotamide	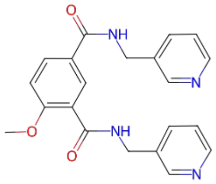	5% (30 µM)	2% (30 µM)
Tucatinib	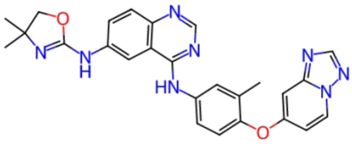	51.45	18.62
Rimegepant	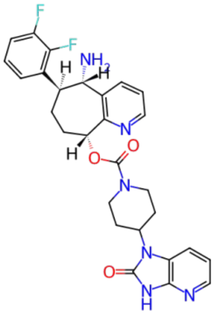	5% (30 µM)	8% (30 µM)
Dasabuvir	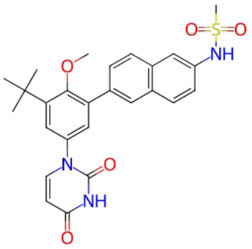	2% (30 µM)	5% (30 µM)
Baricitinib	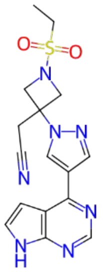	0.019	0.011

Nialamide	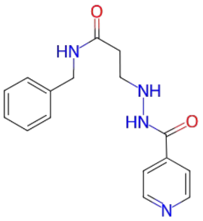	18.8	29.2
Cilostazole	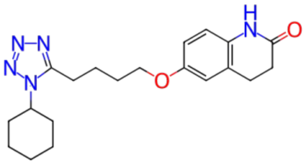	3% (30 µM)	8% (30 µM)
Ponatinib	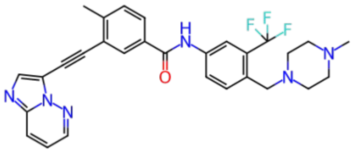	0.197	0.056
Raltegravir	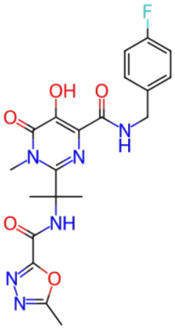	18% (30 µM)	19% (30 µM)
Erdafitinib	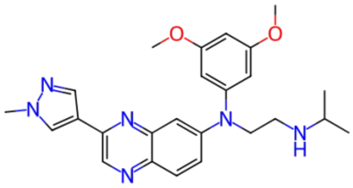	9% (30 µM)	27% (30 µM)
Ruxolitinib	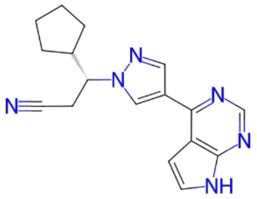	0.025	0.007
Tivozanib	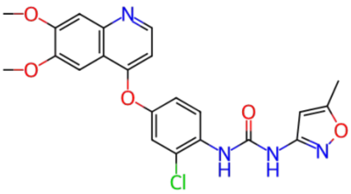	12.86	0.856

Brexpiprazole	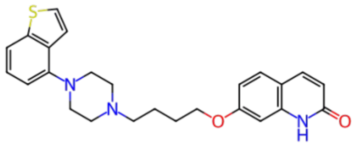	23% (30 µM)	33% (30 µM)
Doravirine	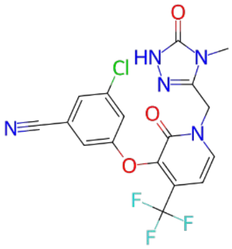	5% (30 µM)	3% (30 µM)
Sertindole	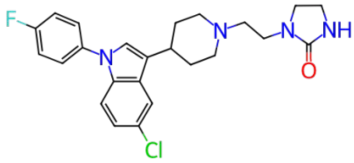	0% (30 µM)	7% (30 µM)

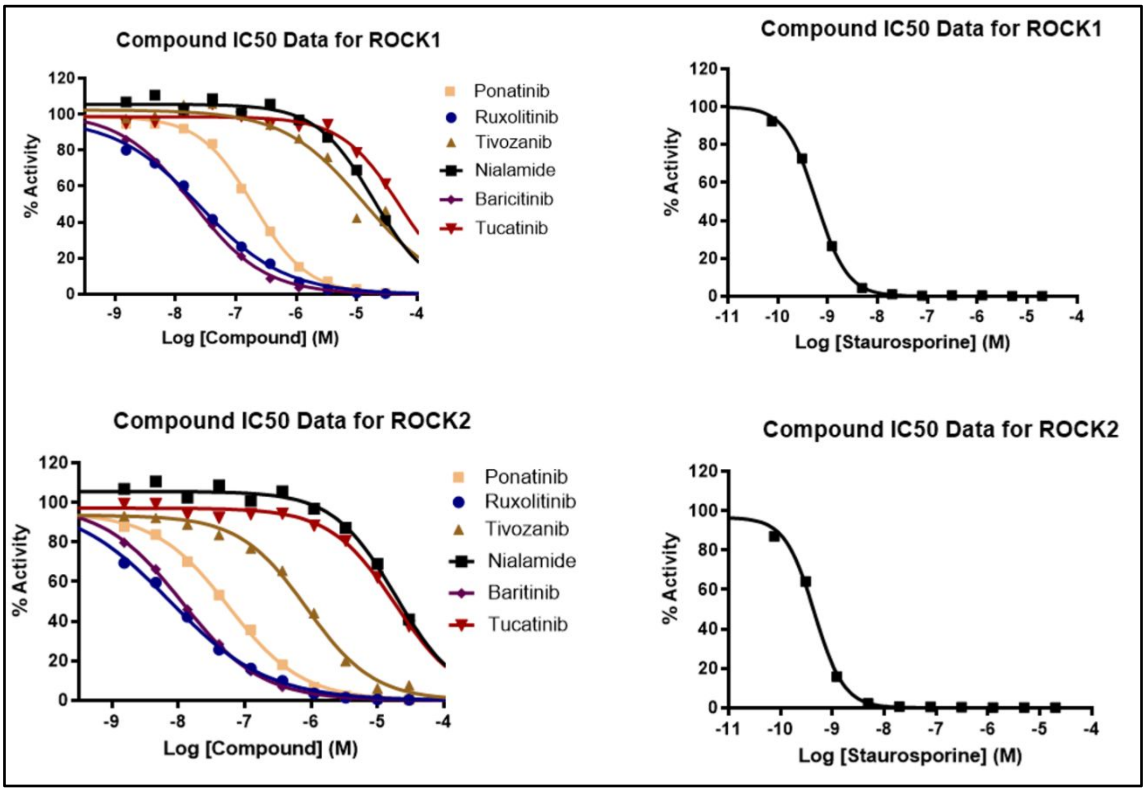

Figure S1. IC_{50} curves for ROCK1 and ROCK2 inhibition.

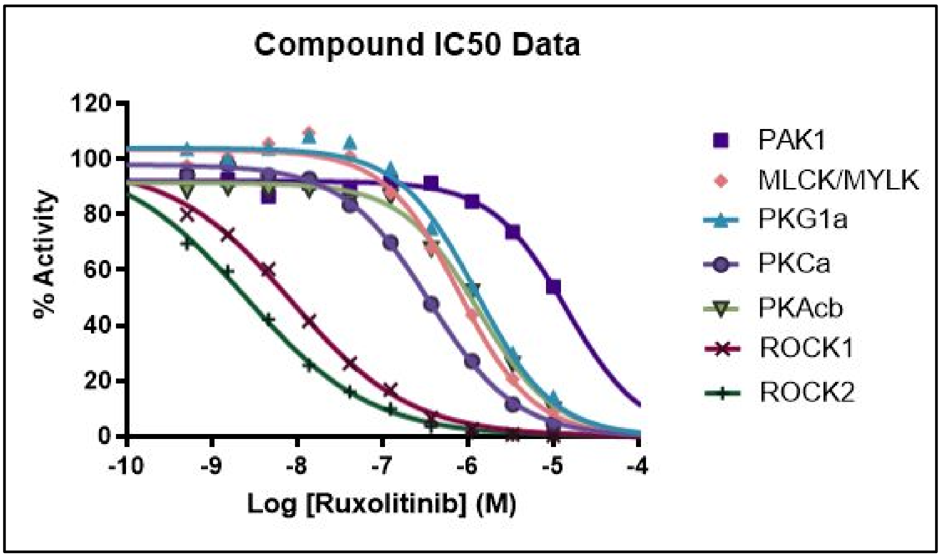

Figure S2. Ruxolitinib's IC_{50} curves for selected kinases of the AGC family.

Analysis Report

SHIMADZU LabSolutions

<Sample Information>

Sample Name	:Ruxolitinib		
Sample ID	:Ruxolitinib		
Data Filename	:BMJRUXOLITINIB-1.lcd		
Method Filename	: 70(C)_30(D)_1p2_254_20min.lcm		
Batch Filename	:		
Vial #	: 1-1	Sample Type	: Unknown
Injection Volume	: 20 uL		
Date Acquired	: 18/03/2025 20:15:02	Acquired by	: System Administrator
Date Processed	: 18/03/2025 20:30:04	Processed by	: System Administrator

<Chromatogram>

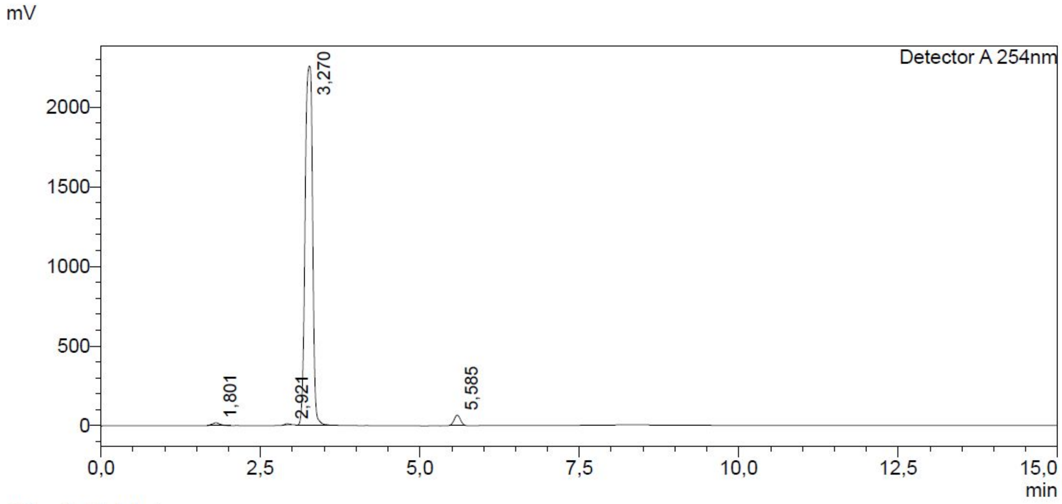

<Peak Table>

Detector A 254nm

Peak#	Ret. Time	Area	Height	Conc.	Unit	Mark	Name
1	1,801	148357	17978	0,782		M	
2	2,921	32691	6684	0,172		M	
3	3,270	18374818	2253903	96,809		M	
4	5,585	424587	63195	2,237		M	
Total		18980454	2341760				

Figure S3. Chromatogram of ruxolitinib obtained using a Shimadzu LC-20AT system equipped with a Thermo Scientific Hypersil BDS C18 column (4.6 mm × 250 mm) and a Shimadzu SPD-20AV detector. The analysis was performed in isocratic mode with an acetonitrile/water (7:3) solvent system at a flow rate of 1.0 mL/min. Ruxolitinib showed a retention time of 3.270 minutes and a purity of 96.8%.

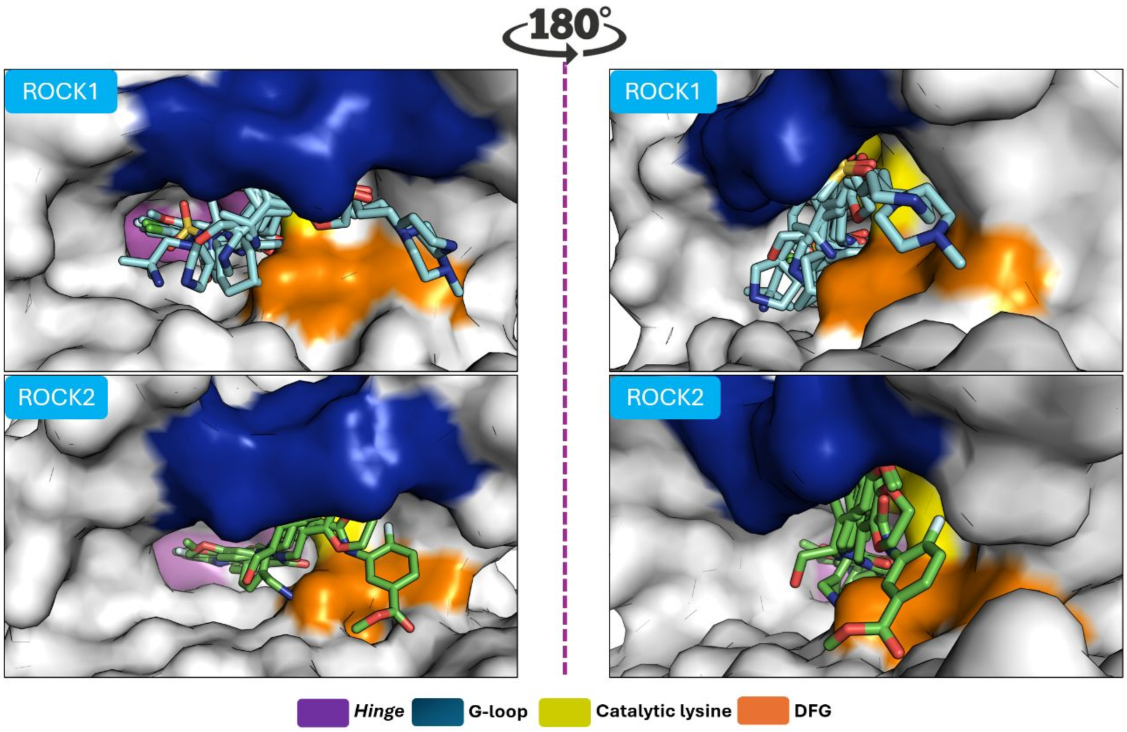

Figure S4. Overlay of selected ROCK1 and ROCK2 crystal structures focusing on the ATP-binding site. ROCK1 ligands are shown with cyan-colored carbon atoms, while ROCK2 ligands have green-colored carbon atoms. Both ROCK1 and ROCK2 are displayed with surfaces where specific regions are highlighted in distinct colors: hinge region (purple), catalytic lysine (yellow), G-loop (blue), and DFG motif (orange).

Table S4. MM/GBSA data obtained from ruxolitinib's MD trajectories in ROCK1 and ROCK2.

Entry	Total ΔG (kcal/mol)	VDWAALS	EEL	EGB	ESURF
ROCK1 – RUN1	-31.01	-43.91	-6.18	24.16	-5.07
ROCK1 – RUN2	-31.11	-42.97	-10.59	27.52	-5.07
ROCK1 – RUN3	-30.16	-43.73	-13.90	32.75	-5.28
ROCK2 – RUN1	-31.71	-46.65	-11.30	31.64	-5.40
ROCK2 – RUN2	-27.53	-37.84	-7.17	21.90	-4.42
ROCK2 – RUN3	-29.54	-46.16	-13.95	36.05	-5.48

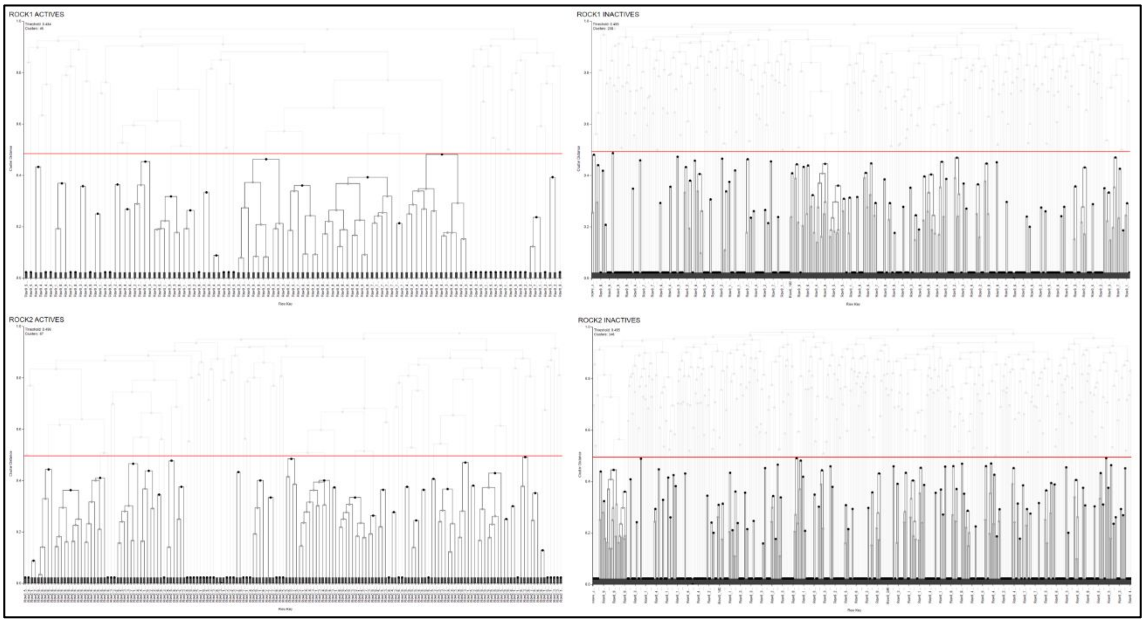

Figure S5. Hierarchical clustering of active and inactive compounds for ROCK1 and ROCK2 based on circular fingerprint similarity. The analysis was performed in KNIME using a distance matrix and a distance threshold of 0.5 to eliminate highly similar compounds and reduce bias in pharmacophoric map validation. This threshold resulted in 45 clusters for ROCK1 actives, 67 for ROCK2 actives, 238 for ROCK1 inactives, and 346 for ROCK2 inactives.
